# Long-Term Estrogen Receptor Beta Agonist Treatment Modifies the Hippocampal Transcriptome in Middle-Aged Ovariectomized Rats

**DOI:** 10.3389/fncel.2016.00149

**Published:** 2016-06-10

**Authors:** Miklós Sárvári, Imre Kalló, Erik Hrabovszky, Norbert Solymosi, Annie Rodolosse, Zsolt Liposits

**Affiliations:** ^1^Laboratory of Endocrine Neurobiology, Institute of Experimental Medicine, Hungarian Academy of SciencesBudapest, Hungary; ^2^Faculty of Information Technology and Bionics, Pázmány Péter Catholic UniversityBudapest, Hungary; ^3^Faculty of Veterinary Science, Szent István UniversityBudapest, Hungary; ^4^Functional Genomics Core, Institute for Research in BiomedicineBarcelona, Spain

**Keywords:** hippocampus, rat, ovariectomy, estrogen receptor β, microarray, PCR, transcriptome, pathway analysis

## Abstract

Estradiol (E2) robustly activates transcription of a broad array of genes in the hippocampal formation of middle-aged ovariectomized rats via estrogen receptors (ERα, ERβ, and G protein-coupled ER). Selective ERβ agonists also influence hippocampal functions, although their downstream molecular targets and mechanisms are not known. In this study, we explored the effects of long-term treatment with ERβ agonist diarylpropionitrile (DPN, 0.05 mg/kg/day, sc.) on the hippocampal transcriptome in ovariectomized, middle-aged (13 month) rats. Isolated hippocampal formations were analyzed by Affymetrix oligonucleotide microarray and quantitative real-time PCR. Four hundred ninety-seven genes fulfilled the absolute fold change higher than 2 (FC > 2) selection criterion. Among them 370 genes were activated. Pathway analysis identified terms including glutamatergic and cholinergic synapse, RNA transport, endocytosis, thyroid hormone signaling, RNA degradation, retrograde endocannabinoid signaling, and mRNA surveillance. PCR studies showed transcriptional regulation of 58 genes encoding growth factors (*Igf2, Igfb2, Igf1r, Fgf1, Mdk, Ntf3, Bdnf*), transcription factors (*Otx2, Msx1*), potassium channels (*Kcne2*), neuropeptides (*Cck, Pdyn*), peptide receptors (*Crhr2, Oprm1, Gnrhr, Galr2, Sstr1, Sstr3*), neurotransmitter receptors (*Htr1a, Htr2c, Htr2a, Gria2, Gria3, Grm5, Gabra1, Chrm5, Adrb1*), and vesicular neurotransmitter transporters (*Slc32a1, Slc17a7*). Protein-protein interaction analysis revealed networking of clusters associated with the regulation of growth/troph factor signaling, transcription, translation, neurotransmitter and neurohormone signaling mechanisms and potassium channels. Collectively, the results reveal the contribution of ERβ-mediated processes to the regulation of transcription, translation, neurogenesis, neuromodulation, and neuroprotection in the hippocampal formation of ovariectomized, middle-aged rats and elucidate regulatory channels responsible for DPN-altered functional patterns. These findings support the notion that selective activation of ERβ may be a viable approach for treating the neural symptoms of E2 deficiency in menopause.

## Introduction

17β-estradiol (E2) exerts profound effects in the brain including the hippocampus (Gould et al., 1990). E2 increases dendritic spine density of principal neurons during the estrous cycle (Woolley et al., 1990), modulates both excitatory (Woolley et al., 1997) and inhibitory (Murphy et al., 1998) neurotransmission, enhances long-term potentiation (LTP) (Foy et al., 1999), regulates neurogenesis (Tanapat et al., 1999), and attenuates the innate immune response (Vegeto et al., 2003). E2 improves cognitive performance in adult and middle-aged ovariectomized (OVX) rats (Talboom et al., 2008; Rodgers et al., 2010) and mice (Walf et al., 2008). These effects are mediated by the two canonical intracellular receptors ERα, ERβ, and the membrane G protein-coupled estrogen receptor GPER, which are expressed in the hippocampal formation (Shughrue and Merchenthaler, 2000; Brailoiu et al., 2007; Mitterling et al., 2010; Waters et al., 2015). The complex action of these receptors results in sensitive responses of the female hippocampus to the cyclic changes of serum E2 levels.

ERα and ERβ mediate the effects of E2 on both intracellular signaling and gene transcription. They share similar domain organization, and use almost identical DNA binding elements, co-regulators, and transcription machinery (Gronemeyer et al., 2004). The expression of the two receptors is not overlapping (Shughrue et al., 1997), and differences in learning and memory exist between ER knockout (ERKO) mice (Han et al., 2013). ERαKO mice show reduced estrogen responsiveness and impaired hippocampus-dependent memory (Foster et al., 2008). ERβKO mice display attenuated hippocampal CA1 LTP and related memory deficits (Day et al., 2005). The phenotypes suggest distinct roles of ERα and ERβ in estrogen signaling.

Combinations of genetic and pharmacological approaches have been successfully used to dissect the role of ERβ in the regulation of hippocampal functions. Activation of ERβ with a selective agonist WAY-200070 enhances LTP and synaptic plasticity (Liu et al., 2008). E2 improves hippocampus-dependent memory in wild type and ERαKO mice, but not in ERβKO animals (Liu et al., 2008). Diarylpropionitrile (DPN) improves cognitive performance in wild type, but not in ERβKO mice (Walf et al., 2008). In rats, behavioral studies have shown that DPN evokes anxiolytic effects (Walf and Frye, 2005) and enhances performance in hippocampus-dependent memory tasks (Rhodes and Frye, 2006; Pisani et al., 2016). These studies suggest that ERβ agonists mimic many basic effects of E2 in the hippocampus and enhance hippocampus-dependent spatial memory (Rhodes and Frye, 2006; Jacome et al., 2010; Pisani et al., 2012, 2016).

Human menopause is characterized by declining levels of E2 that influence the performance of the hippocampal formation (Weber et al., 2013). Symptoms may include anxiety, mild depression, disturbances in contextual fear extinction, impairment of spatial memory and regression of sexual behavior. We have shown that in 13 months old OVX rats the hippocampal transcriptome is highly responsive to timely E2 replacement (Sarvari et al., 2015). In rodent menopause models, ERβ agonists decrease anxiety (Walf and Frye, 2005; Hughes et al., 2008) and depression (Clark et al., 2012; Suzuki et al., 2013), improve memory (Jacome et al., 2010; Kiss et al., 2012), and modulate fear extinction (Chang et al., 2009; Zeidan et al., 2011). Hence, development of selective ERβ agonists may result in safe and selective practice of hormone replacement therapy. The present study was devoted to the aforementioned concept with the main task to determine whether long-term DPN treatment of middle-aged OVX rats has an impact on the hippocampal transcriptome and to elucidate the putative cellular mechanisms and regulatory channels involved.

## Materials and methods

### Experimental animals

All experiments were performed with permission from the Animal Welfare Committee of the Institute of Experimental Medicine (IEM, Permission Number: A5769-01) and in accordance with regulations of the European Community (Decree 86/609/EEC). Female Harlan-Wistar rats were purchased from Toxicoop (Budapest, Hungary) and housed on a 12 h light/12 h dark cycle in the animal care facility of IEM. The rats were used as breeders and retired at their age of 8 months, and then housed individually for the subsequent months. At their age of 13 months, rats were deeply anesthetized and ovariectomized bilaterally (Sarvari et al., 2010). Afterward, they were kept on phytoestrogen-free diet (Harlan Teklad Global Diets, Madison, WI). Ten days later, Alzet 2004 osmotic minipumps (DURECT, Cupertino, CA) filled either with DPN (3.3 mg/ml in propylene-glycol, *n* = 8, DPN group) or vehicle only (*n* = 8, control group) were implanted subcutaneously in the scruff of the neck of the animals. This treatment lasted for 29 days providing a dosage of 0.02 mg DPN/animal/day. The same animals and hippocampal samples have been used in a recent PCR study (Sarvari et al., 2014).

### Affymetrix rat genome 230 PM strip arrays

Hippocampal formations from 16 animals were prepared and total RNA was isolated and analyzed as described previously (Sarvari et al., 2014). RNA quality was measured by capillary electrophoresis using 2100 Agilent Bioanalyzer (Santa Clara, CA, USA) with Nano RNA chips. RNA samples displayed high RNA integrity numbers (RIN > 8.2). Eight samples were examined by oligonucleotide microarray, including amplification, target labeling, hybridization, staining, and scanning steps, which were carried out as described earlier (Sarvari et al., 2015). In brief, 25 ng of total RNA Whole Transcriptome Amplification (WTA) library preparation and amplification for 17 cycles were performed following distributor's (Sigma-Aldrich) recommendations. Eight micrograms cDNA was fragmented by DNAseI and biotinylated by terminal transferase obtained from the GeneChip Mapping 250K Nsp Assay Kit (Affymetrix Inc., Santa Clara, CA, USA). Hybridization, washing, staining and scanning of Affymetrix Rat Genome 230 PM Strip arrays were performed following the manufacturer's recommendations. Scanned images (DAT files) were transformed into intensities (CEL files) using the AGCC software (Affymetrix). Data analysis, including GCRMA, statistical and data mining work, were carried out as published earlier (Sarvari et al., 2015). Gene Ontology (GO) term enrichment was analyzed using public functional annotation tools [DAVID Bioinformatics Resources; http://david.abcc.ncifcrf.gov (Huang Da et al., 2009), and KEGG pathway database; http://www.genome.jp/kegg]. Annotation clusters were ranked by their score number, termed enrichment score, calculated from the modified Fisher's exact *p*-value of each GO-term. Putative protein-protein interactions were evaluated by the web-based STRING 10 platform (http://string-db.org).

### Quantitative real-time PCR

Custom TaqMan microfluidic cards (Applied Biosystems, Foster City, CA, USA) were designed to study mRNA expression by real-time PCR. Sixteen samples were examined. One microgram of total RNA was used for reverse transcription. Reverse transcription and PCR were carried out by using Applied Biosystems' High Capacity cDNA Reverse Transcription Kit and TaqMan Universal PCR Master Mix II, respectively. The ViiA7 RUO 1.2.1 (Applied Biosystems) software and relative quantification against calibrator samples (ΔΔCt) were used for data evaluation. Glyceraldehyde-3-phosphate dehydrogenase (*Gapdh*) and hypoxanthine guanine phosphoribosyl-transferase (*Hprt*) were used as housekeeping genes. Expression of these genes did not vary among experimental groups. A computed internal control corresponding to the geometric mean of cycle threshold (Ct) values of *Gapdh* and *Hprt* was used for Ct calculation. PCR data evaluation were performed as described previously (Sarvari et al., 2014).

## Results

### Effect of long-term DPN administration on uterus weight

Middle-aged, OVX rats received DPN at a dose of 20 μg/day, subcutaneously via osmotic minipump for 29 days. At the end of treatment, the potential proliferative threat on the uterus was checked. The uterus weight of vehicle- and ERβ agonist-treated rats were 205 ± 43.2 and 163 ± 30.0 mg, respectively, indicating that DPN at this dose and treatment time did not induce cell proliferation in the uterus.

### Changes of the hippocampal transcriptome in response to chronic DPN treatment

#### Microarray study

Differential expression was studied in the hippocampal formation by comparing vehicle- and ERβ agonist-treated animals. We found that chronic treatment with DPN evoked significant changes in the transcriptome. We considered a gene regulated if the change of its transcription, i.e., the modulus of its fold change (mFC), exceeded 2. Symbols and description of regulated genes are listed in Supplementary Table 1. Four hundred ninety-seven genes fulfilled the mFC > 2.0 selection criterion. From these, 370 were activated (Supplementary Table 2).

The top 59 upregulated and 11 downregulated genes, which satisfied the mFC > 2.7 criterion, were listed in Table 1. DPN robustly activated (FC > 4) the transcription of 18 genes including transthyretin (*Ttr*), claudin 2 (*Cldn2*), klotho (*Kl*), insulin-like growth factor-binding protein 2 (*Igfbp2*), secretoglobin (*Scgb1c1*), folate (*Folr1*), and prolactin (*Prlr*) receptors, sclerostin domain containing (*Sostdc1*), small nuclear ribonucleoprotein polypeptide G (*Snrpg*), prostaglandin D2 synthase (*Ptgds*), insulin-like growth factor 2 (*Igf2*), malectin (*Mlec*), SEC63 homolog (*Sec63*), membrane frizzled-related protein (*Mfrp*), spectrin beta, non erythrocytic 4 (*Sptbn4*), midkine (*Mdk*), angiotensin I converting enzyme (*Ace*), and poliovirus related receptor 1 (*Pvrl*). The single robustly suppressed target gene (FC < 0.25) was stem-loop binding protein (*Slbp*).

**Table 1:** **List of DPN-regulated genes in the hippocampus**.

**Probeset ID**	**LogFC**	**FC**	***P* Adjusted**	**Symbol**	**Description**
**UPREGULATED GENES**					
1367598_PM_at	4.863	29.104	0.005	*Ttr*	Transthyretin
1375933_PM_at	3.991	15.895	0.013	*Cldn2*	Claudin 2
1369361_PM_at	3.143	8.834	0.031	*Kl*	Klotho
1367648_PM_at	3.105	8.602	0.015	*Igfbp2*	Insulin-like growth factor binding protein 2
1393436_PM_at	2.852	7.219	0.017	*Scgb1c1*	Secretoglobin, family 1C, member 1
1387889_PM_at	2.730	6.635	0.014	*Folr1*	Folate receptor 1 (adult)
1370789_PM_a_at	2.670	6.366	0.018	*Prlr*	Prolactin receptor
1379281_PM_at	2.640	6.235	0.031	*Sostdc1*	Sclerostin domain containing 1
1372167_PM_at	2.325	5.009	0.008	*Snrpg*	Small nuclear ribonucleoprotein poly peptide G
1367851_PM_at	2.313	4.968	0.007	*Ptgds*	Prostaglandin D2 synthase (brain)
1367571_PM_a_at	2.305	4.940	0.032	*Igf2*	Insulin-like growth factor 2
1379046_PM_at	2.279	4.854	0.013	*Mlec*	Malectin
1384519_PM_at	2.185	4.547	0.006	*Sec63*	SEC63 homolog (*S. cerevisiae*)
1377434_PM_at	2.173	4.509	0.047	*Mfrp*	Membrane frizzled-related protein
1376564_PM_at	2.136	4.397	0.006	*Sptbn4*	Spectrin, beta, non-erythrocytic 4
1367682_PM_at	2.068	4.193	0.013	*Mdk*	Midkine
1387791_PM_at	2.061	4.173	0.036	*Ace*	Angiotensin I converting enzyme
1388215_PM_at	2.055	4.156	0.007	*Pvrl1*	Poliovirus receptor-related 1
1370844_PM_at	1.913	3.767	0.014	*Hnrnpf*	Heterogeneous nuclear ribonucleoprotein F
1390532_PM_at	1.911	3.760	0.040	*Slc13a4*	Sodium/sulfate symporters
1371959_PM_at	1.891	3.710	0.005	*Hist2h2aa3*	Histone cluster 2, H2aa3
1374320_PM_at	1.838	3.575	0.061	*F5*	Coagulation factor V (proaccelerin)
1380475_PM_at	1.836	3.570	0.005	*Gigyf2*	GRB10 interacting GYF protein 2
1380549_PM_at	1.833	3.562	0.005	*Ccar1*	Cell division cycle and apoptosis regulator 1
1383386_PM_a_at	1.827	3.548	0.011	*Sec3l1*	SEC3-like 1 (*S. cerevisiae*)
1372893_PM_at	1.746	3.354	0.008	*Yipf1*	Yip1 domain family, member 1
1378971_PM_at	1.745	3.351	0.005	*Mllt6*	Myeloid/lymphoid or mix ed-lineage leukemia
1377061_PM_at	1.721	3.296	0.007	*Arhgap32*	Rho GTPase activating protein 32
1388244_PM_s_at	1.680	3.204	0.030	*Rpsa*	Ribosomal protein SA
1379469_PM_at	1.669	3.180	0.006	*Tbl1x*	Transducin (beta)-like 1 X-linked
1369568_PM_at	1.658	3.155	0.010	*Stx6*	Syntax in 6
1394343_PM_s_at	1.655	3.150	0.078	*Kcne2*	Potassium voltage-gated channel, Isk-related
1395053_PM_at	1.655	3.149	0.012	*Pds5b*	PDS5, regulator of cohesion maintenance
1381537_PM_at	1.627	3.089	0.006	*Klc3*	Kinesin light chain 3
1390767_PM_at	1.611	3.055	0.006	*Ssr1*	Signal sequence receptor, alpha
1391312_PM_at	1.590	3.010	0.003	*Inpp5f*	Inositol poly phosphate-5-phosphatase F
1382489_PM_at	1.586	3.003	0.009	*Phip*	Pleckstrin homology domain interacting protein
1377997_PM_at	1.577	2.984	0.008	*Agtpbp1*	ATP/GTP binding protein 1
1390017_PM_at	1.571	2.972	0.016	*Hapln2*	Hyaluronan and proteogly can link protein 2
1390677_PM_at	1.571	2.971	0.020	*Fibcd1*	Fibrinogen C domain containing 1
1372916_PM_at	1.570	2.970	0.010	*Mrps27*	mitochondrial ribosomal protein S27
1388073_PM_a_at	1.561	2.950	0.010	*Nupl1*	Nucleoporin like 1
1376533_PM_at	1.536	2.900	0.010	*Baz2b*	Bromodomain adjacent to zinc finger domain, 2B
1383418_PM_at	1.528	2.883	0.008	*Adam11*	ADAM metallopeptidase domain 11
Affx _Rat_Hex _3_at	1.527	2.881	0.014	*Hk1*	Hexokinase 1
1381728_PM_at	1.501	2.830	0.005	*Prkag2*	Protein kinase, AMP-activ ated, gamma 2 subunit
1370800_PM_at	1.499	2.826	0.009	*Slc9a1*	Sodium/hydrogen exchanger
1379329_PM_at	1.498	2.824	0.010	*Pcbd2*	Pterin 4 alpha carbinolamine dehydratase
1393325_PM_at	1.484	2.797	0.006	*Gtpbp6*	GTP binding protein 6 (putative)
1375149_PM_at	1.483	2.796	0.005	*Lrrc4b*	Leucine rich repeat containing 4B
1395913_PM_at	1.482	2.794	0.006	*Acbd4*	Acy l-CoA binding domain containing 4
1371335_PM_at	1.481	2.792	0.010	*Atp5l*	ATP synthase, H+ transporting, mitochondrial Fo
1388568_PM_at	1.476	2.782	0.024	*Eif3d*	Eukaryotic translation initiation factor 3, subunit
1374172_PM_at	1.474	2.778	0.049	*Col8a2*	Collagen, type VIII, alpha 2
1368729_PM_a_at	1.451	2.734	0.009	*Adcyap1r1*	Adenylate cyclase activating poly peptide 1 receptor
1388396_PM_at	1.450	2.731	0.007	*Stk25*	Serine/threonine kinase 25
1398487_PM_at	1.443	2.719	0.006	*Pbx1*	Pre-B-cell leukemia homeobox 1
1388531_PM_at	1.442	2.717	0.024	*Pgrmc2*	Progesterone receptor membrane component 2
1383920_PM_at	1.442	2.716	0.005	*Amt*	Aminomethyltransferase
**DOWNREGULATED GENES**					
1398355_PM_at	−1.449	0.366	0.025	*Trpm7*	Transient receptor potential cation channel, subfamily M
1371243_PM_at	−1.451	0.366	0.011	*Grm8*	Glutamate receptor, metabotropic 8
1383192_PM_at	−1.452	0.366	0.006	*Spast*	Spastin
1388755_PM_at	−1.461	0.363	0.011	*Sec23a*	Sec23 homolog A (*S. cerevisiae*)
1395461_PM_at	−1.493	0.355	0.005	*Apc2*	Adenomatosis polyposis coli 2
1383172_PM_at	−1.507	0.352	0.007	*Ranbp2*	RAN binding protein 2
1389479_PM_at	−1.569	0.337	0.009	*Klf3*	Kruppel-like factor 3 (basic)
1381859_PM_at	−1.607	0.328	0.018	*Fam91a1*	Family with sequence similarity 91, member A1
1388689_PM_at	−1.682	0.312	0.007	*Acyp2*	Acylphosphatase 2, muscle type
1390509_PM_a_at	−1.913	0.265	0.013	*Mrpl51*	Mitochondrial ribosomal protein L51
1391387_PM_s_at	−2.186	0.220	0.020	*Slbp*	Stem-loop binding protein

*Expression profiling using Affymetrix oligonucleotide microarray revealed that DPN regulates the hippocampal transcriptome in middle-aged, OVX rats. Using the fold change >2.7 selection criterion, the list of microarray data contains 59 up- and 11 downregulated genes*.

#### Heat map analysis

**Figure 1:**
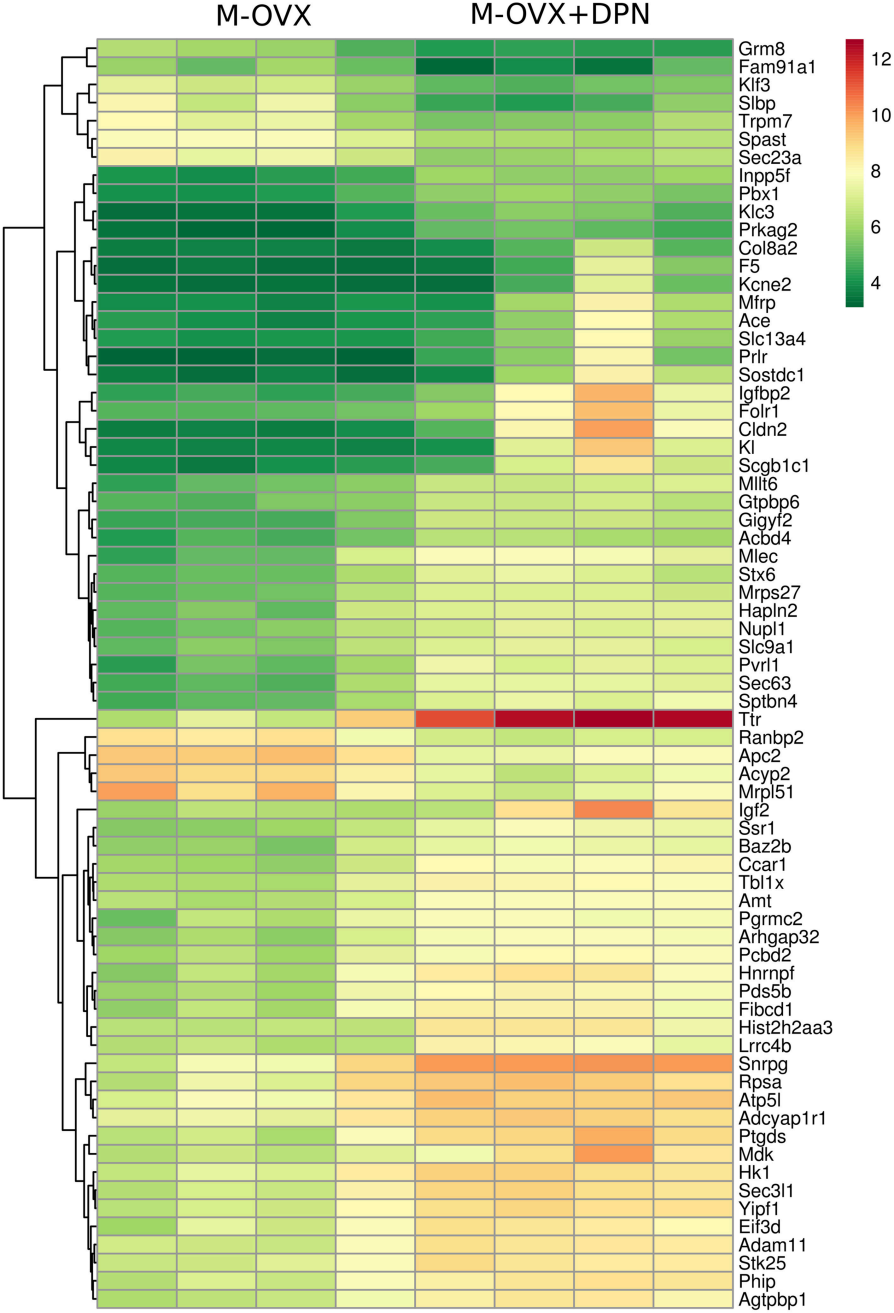
**Heat map**. It depicts 70 differentially expressed genes in the hippocampus of middle-aged OVX rats treated for 29 days with vehicle or ERβ agonist DPN, respectively. Rows represent DPN-regulated probe sets with corresponding gene symbols displayed on the right side of the figure. Transcription level of each probe is color coded, the continuous color key is displayed in the upper right corner. Individual hippocampal samples of four vehicle (M-OVX)- and four DPN (M+OVX+DPN)-treated animals are shown in columns.

The heat map represents mRNA expression of the top 70 differentially expressed genes in the hippocampal formation of vehicle treated (M-OVX) and DPN-treated (M-OVX+DPN) animals (Figure 1). The heat map demonstrated that the hippocampal transcriptome robustly responded to long-term DPN treatment. The majority of transcriptional changes was activation. The color-coded visualization of mRNA expression levels displayed only small variability within the experimental groups.

#### Functional annotation of DPN-regulated genes

Pathway analysis and functional annotation clustering of DPN-regulated genes were performed on web-based platforms, KEGG, and DAVID v6.7 bioinformatics tool, respectively. KEGG analysis (Table 2) identified terms related to neurotransmission including glutamatergic synapse (*Gng12, Dlgap1, Cacna1a, Shank2, Gls, Gnbr, Kcnj3, Grm1, Grm8*), cholinergic synapse (*Chrm4, Gng12, Pik3ca, Fos, Cacna1a, Chrm1, Gnbr, Kcnj3*), RNA transport (*Tpr, Srrm1, Eif3d, Eif4g1, Thoc2, Eif3e, Upf3a, Nupl1, Pabpc1, Ranbp2*), endocytosis, thyroid hormone signaling pathway (*Hif1a, Pik3ca, Med1, Ncor1, Slc9a1, Crebbp, Pfkfb2, Atp2a2*), RNA degradation (*Dhx36, Cnot6l, Edc4, Pabpc1, Ddx6, Lsm7*), retrograde endocannabinoid signaling (*Gng12, Cacna1a, Mgll, Mapk10, Gnbr, Kcnj3, Grm1*), and mRNA surveillance pathway (*Papolg, Pabpn1, Srrm1, Upf3a, Cpsf3, Pabpc1*). By the use of STRING program, putative interactions of certain proteins encoded by the DPN-regulated genes, obtained from the KEGG pathway analysis, were revealed and plotted (Supplementary Figure 1).

**Table 2:** **Pathway analysis**.

**GO ID**	**GO terms and corresponding genes**	**Gene no**.	***P***	**P_fdr**
4724	**Glutamatergic synapse**	9	4.68E-04	1.33E-01
	Gng12, Dlgap1, Cacna1a, Shank2, Gls, Gnb5, Kcnj3, Grm1, Grm8			
4725	**Cholinergic synapse**	8	1.51E-03	1.52E-01
	Chrm4, Gng12, Pik3ca, Fos, Cacna1a, Chrm1, Gnb5, Kcnj3			
3013	**RNA transport**	10	1.86E-03	1.52E-01
	Tpr, Srrm1, Eif3d, Eif4g1, Thoc2, Eif3e, Upf3a, Nupl1, Pabpc1, Ranbp2			
4144	**Endocytosis**	12	2.11E-03	1.52E-01
	Asap1, Epn2, Arfgap1, Folr1, Vps45, Cxcr4, Nedd4, RT1-A1, Sh3gl2, Erbb4, Grk6, Vps4a			
4919	**Thyroid hormone signaling pathway**	8	2.87E-03	1.63E-01
	Hif1a, Pik3ca, Med1, Ncor1, Slc9a1, Crebbp, Pfkfb2, Atp2a2			
3018	**RNA degradation**	6	4.68E-03	1.92E-01
	Dhx36, Cnot6l, Edc4, Pabpc1, Ddx6, Lsm7			
4723	**Retrograde endocannabinoid signaling**	7	4.69E-03	1.92E-01
	Gng12, Cacna1a, Mgll, Mapk10, Gnb5, Kcnj3, Grm1			
3015	**mRNA surveillance pathway**	8	1.07E-02	3.84E-01
	Papolg, Pabpn1, Srrm1, Upf3a, Cpsf3,Pabpc1			

*Top GO terms of genes expressed differentially in the hippocampus of middle-aged OVX female rats treated with ERβ agonist DPN or vehicle as control. The analysis was performed on the web-based KEGG platform. Terms were ranked based on their fdr values. Genes are listed at each GO term. Fdr, false discovery rate*.

Another functional annotation tool (DAVID) identified 12 clusters with high enrichment score (ES), such as membrane-enclosed lumen (ES = 4.39), mRNA metabolic process (3.16), protein transport (2.24), nuclear mRNA splicing (2.18), regulation of transcription (1.92), fatty acid transport (1.47), regulation of carbohydrate metabolism (1.45), nucleotide binding (1.44), potassium channel complex (1.41), macromolecular complex assembly (1.41), vesicle coating (1.39), and DNA-dependent transcription (1.3) clusters (Supplementary Table 3).

#### Quantitative real-time PCR study of DPN-regulated genes

We applied quantitative real-time PCR to confirm DPN-dependent regulation of selected genes and to get further insight to the impact of DPN on neurotransmission. We selected 21 genes from the microarray gene list to confirm regulation and 37 additional genes which are associated with neurotransmitter and neuropeptide signaling. Selected genes and changes in their mRNA expression are summarized in Table 3. The PCR study revealed robustly and moderately regulated genes. Robustly activated genes (RQ > 4.0; 17 genes) included *Sostdc1, Ttr, Kl, Prlr, Slco1a5, Mfrp, Aqp1, Otx2, Cldn2, Igfbp2, Folr1, Igf2, Ace, Crhr2, Htr2c, Slc13a4*, and *Enpp2*. Group of moderately activated genes (4 > RQ > 1.2; 28 genes) contained *Cdkn1c, Kcne2, Cox8b, Fgf1, Mdk, Sfrp1, Msx1, Anxa2, Ptgds, A2m, Aldh1a2, Mrc1, Slc12a2, Cdkn1a, Htr2a, Gpx1, Gria2, Oprm1, Igf1r, Inpp5f*, *Ntf3, Gnrhr, Chrm5, Nr3c1, Grm5, Gria3, Gphn, Gabra1*. The group of downregulated genes coded for neuropeptides (*Pdyn, Cck*), neuropeptide receptors (*Sstr1, Sstr3, Galr2*), neurotransmitter receptors (*Htr1a, Adrb1*), vesicular transporters (*Slc17a7, Slc32a1*), neurotrophic factor (*Bdnf*), and others (*Gad1, Apoe, Atp2b4*). Importantly, DPN-dependent regulation of the 21 selected genes including *Sostdc1, Ttr, Kl, Prlr, Slco1a5, Mfrp, Aqp1, Otx2, Cldn2, Igfbp2, Folr1, Igf2, Ace, Htr2c, Slc13a4, Enpp2, Kcne2, Mdk, Ptgds, Inpp5f*, *A2m* was confirmed by the PCR study.

**Table 3:** **PCR Results**.

**Assay ID**	**RQ**	***P***	**Symbol**	**Gene name**
Rn00596672_m1	182.9	0.034	*Sostdc1*	Sclerostin domain-containing protein 1
Rn00562124_m1	175.1	0.027	*Ttr*	Transthyretin
Rn00580123_m1	141.4	0.040	*Kl*	Klotho
Rn01525459_m1	76.08	0.020	*Prlr*	Prolactin receptor
Rn01463516_m1	60.62	0.036	*Slco1a5*	Organic anion transporter
RN01417754_g1	49.54	0.053	*Mfrp*	Membrane-type frizzled-related protein
Rn00562834_m1	47.95	0.031	*Aqp1*	Aquaporin
Rn01414596_m1	35.65	0.034	*Otx2*	Orthodenticle homeobox 2
Rn02063575_s1	13.32	0.044	*Cldn2*	Claudin 2
Rn00565473_m1	12.44	0.025	*Igfbp2*	Igf binding protein 2
Rn00591759_m1	12.41	0.029	*Folr1*	Folate receptor 1, adult
Rn01454518_m1	8.625	0.057	*Igf2*	Insulin-like growth factor 2
Rn00561094_m1	8.042	0.037	*Ace*	Angiotensin converting enzyme
Rn00575617_m1	7.910	0.069	*Crhr2*	Corticotropin-releasing hormone receptor 2
Rn00562748_m1	7.223	0.061	*Htr2c*	5-Ht (serotonin) receptor 2c
Rn01747911_m1	6.756	0.027	*Slc13a4*	Sodium-dependent dicarboxylate transporter
Rn01505088_m1	4.694	0.027	*Enpp2*	Ectonucleotide pyrophosphatase
Rn01502044_g1	3.793	0.035	*Cdkn1c*	Cyclin-dependent kinase inhibitor 1c
Rn02094913_s1	3.758	0.044	*Kcne2*	Potassium channel, voltage-gated, isk-related
Rn00562884_m1	3.600	0.058	*Cox8b*	Cytochrome c oxidase, subunit VIIIb (Cox8b)
Rn00689153_m1	3.049	0.058	*Fgf1*	Fibroblast growth factor 1
Rn00675549_g1	2.889	0.024	*Mdk*	midkine
Rn01478472_m1	2.777	0.088	*Sfrp1*	Secreted frizzled-related protein 1
Rn00667535_m1	2.412	0.066	*Msx1*	Muscle segment homeobox, drosophila, homolog
Rn00571516_m1	2.293	0.052	*Anxa2*	Annexin a2
Rn00564605_m1	1.919	0.072	*Ptgds*	Prostaglandin D2 synthase, brain
Rn00560589_m1	1.745	0.057	*A2m*	Alpha-2-macroglobulin
Rn00588079_m1	1.626	0.078	*Aldh1a2*	Aldehyde dehydrogenase 1 family, member a2
Rn01487342_m1	1.576	0.065	*Mrc1*	Mannose receptor, c-type, 1
Rn00582505_m1	1.505	0.085	*Slc12a2*	Sodium/potassium/chloride transporter
Rn01427989_s1	1.502	0.036	*Cdkn1a*	Cyclin-dependent kinase inhibitor 1a
Rn00568473_m1	1.440	0.018	*Htr2a*	5-Ht (serotonin) receptor 2a
Rn00577994_g1	1.368	0.039	*Gpx1*	Glutathione peroxidase
Rn00568514_m1	1.338	0.007	*Gria2*	Glutamate receptor, ionotropic, ampa 2
Rn01430371_m1	1.293	0.029	*Oprm1*	Opioid receptor, mu-1
Rn00583837_m1	1.265	0.055	*Igf1r*	Insulin-like growth factor 1 receptor
Rn01508975_m1	1.252	0.066	*Inpp5f*	Inositol polyphosphate 5-phosphatase f
Rn00579280_m1	1.243	0.045	*Ntf3*	Neurotrophin 3
Rn00578981_m1	1.241	0.049	*Gnrhr*	GnRH receptor
Rn02758749_s1	1.237	0.052	*Chrm5*	Cholinergic receptor, muscarinic 5
Rn00561369_m1	1.236	0.074	*Nr3c1*	Nuclear receptor subfamily 3, group c, member 1
Rn00566628_m1	1.228	0.041	*Grm5*	Glutamate receptor, metabotropic 5
Rn00583547_m1	1.227	0.004	*Gria3*	Glutamate receptor, ionotropic, ampa 3
Rn00575867_m1	1.214	0.041	*Gphn*	Gephyrin
Rn00788315_m1	1.208	0.091	*Gabra1*	GABA a receptor, subunit alpha 1
Rn00563215_m1	0.801	0.033	*Cck*	Cholecystokinin
Rn00571351_m1	0.791	0.050	*Pdyn*	Prodynorphin
Rn00561409_s1	0.773	0.018	*Htr1a*	5-Ht (serotonin) receptor 1a
Rn00824536_s1	0.756	0.034	*Adrb1*	Beta-1-adrenergic receptor
Rn00593680_m1	0.748	0.026	*Apoe*	Apolipoprotein E
Rn01483363_m1	0.720	0.036	*Atp2b4*	Atpase, ca(2+)-transporting, plasma membrane, 4
Rn00690300_m1	0.688	0.010	*Gad1*	67 kda glutamic acid decarboxylase - gad67
Rn00695901_g1	0.661	0.067	*Galr2*	Galanin receptor 2
Rn00824654_m1	0.600	0.002	*Slc32a1*	GABA vesicular transporter
Rn02531967_s1	0.580	0.003	*Bdnf*	Brain-derived neurotrophic factor
Rn02134439_s1	0.576	0.002	*Sstr3*	Somatostatin receptor 3
Rn02532012_s1	0.463	0.029	*Sstr1*	Somatostatin receptor 1
Rn01462431_m1	0.366	0.012	*Slc17a7*	Vesicular glutamate transporter 1 - vglut1

*Real-time PCR study revealed transcriptional regulation of 58 genes. Forty-five of them were upregulated. The DPN regulated genes code for neuropeptides, neurotransmitter and neurohormone receptors, growth hormones, transcription factors, and transporters. RQ, relative quantity*.

### Predicted networking of proteins encoded by DPN-regulated genes

**Figure 2:**
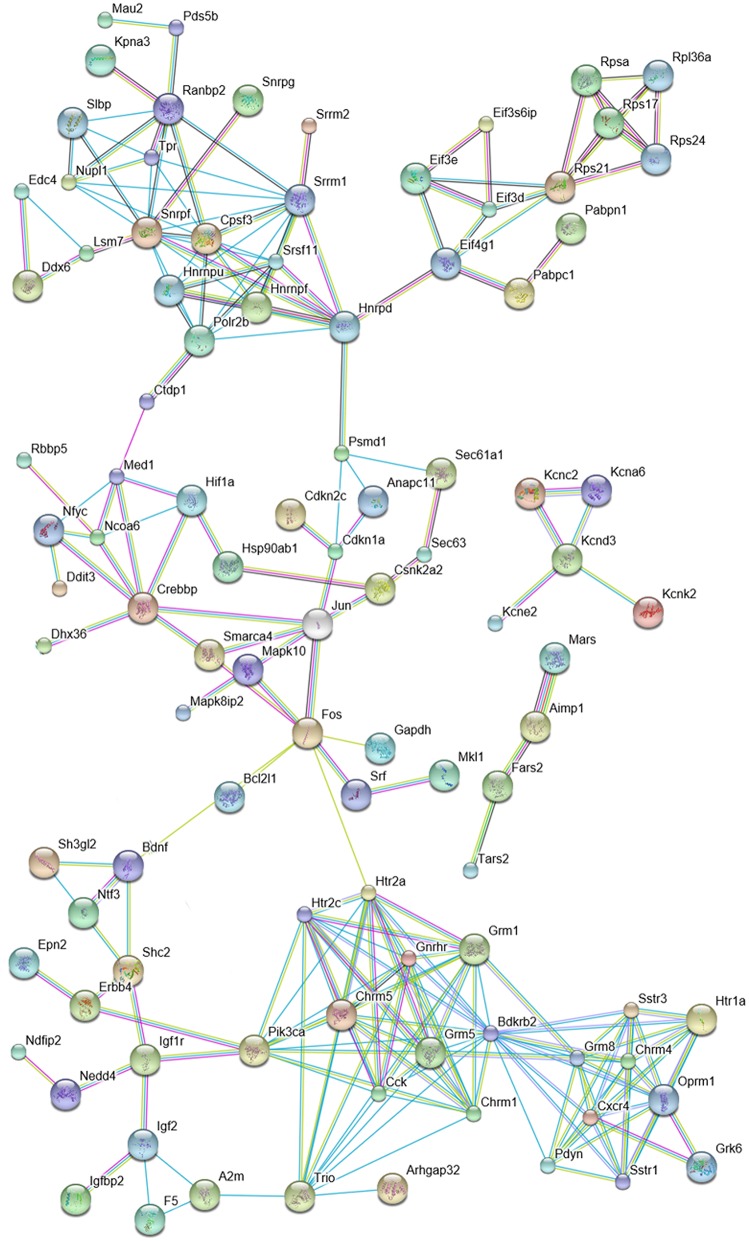
**Predicted interactions among proteins encoded by DPN-regulated genes**. The network is based on the results of microarray and quantitative real-time PCR studies and was constructed by using the STRING 10 Known and Predicted Protein-Protein Interactions program (http://string-db.org/) (see Supplementary Figure 2). Analysis was performed at high confidence value (0.9), non-interacting elements were excluded. Selected protein clusters of the network are depicted. **Cluster 1**: mRNA metabolism, splicing and translation; **Cluster 2**: regulation of transcription (red elliptic line highlights the relationship of Crebbp, Mapk8p2, Mapk10, Fos and Jun); **Cluster 3:** components of potassium channels; **Cluster 4**: growth/troph factors; **Cluster 5**: peptides, neurotransmitter and neuropeptide receptors.

Altogether, we identified 534 DPN-regulated genes by expression profiling. We searched for interactions among proteins encoded by these genes using the STRING 10 platform. At high confidence level (0.72), STRING predicted a large number of putative interactions and clusters composed of more than four elements indicating that DPN may modulate multiple regulatory mechanisms involved in hippocampal functions (Supplementary Figure 2). STRING 10 analysis performed at the highest confidence value (0.9) and filtering clusters for having more than 3 interacting proteins, predicted interactions among 95 proteins (Figure 2). These 95 proteins form functional clusters that may also network with each other. Five major clusters emerged from the analysis. Cluster 1 contained 31 proteins that are associated with transcription, mRNA metabolism, splicing, and translation. This group networks with cluster 2 comprised of 26 intracellular signaling molecules and regulators of transcription. The individual cluster 3 had five potassium channel components. Cluster 4 is linked to cluster 2 and 5, and included 13 growth/troph factors and related proteins. Cluster 5 contained 20, mostly G protein-coupled neurotransmitter and neuropeptide receptors and neuropeptides. This cluster is wired to cluster 4 via Pik3ca (and Trio).

**Figure 3:**
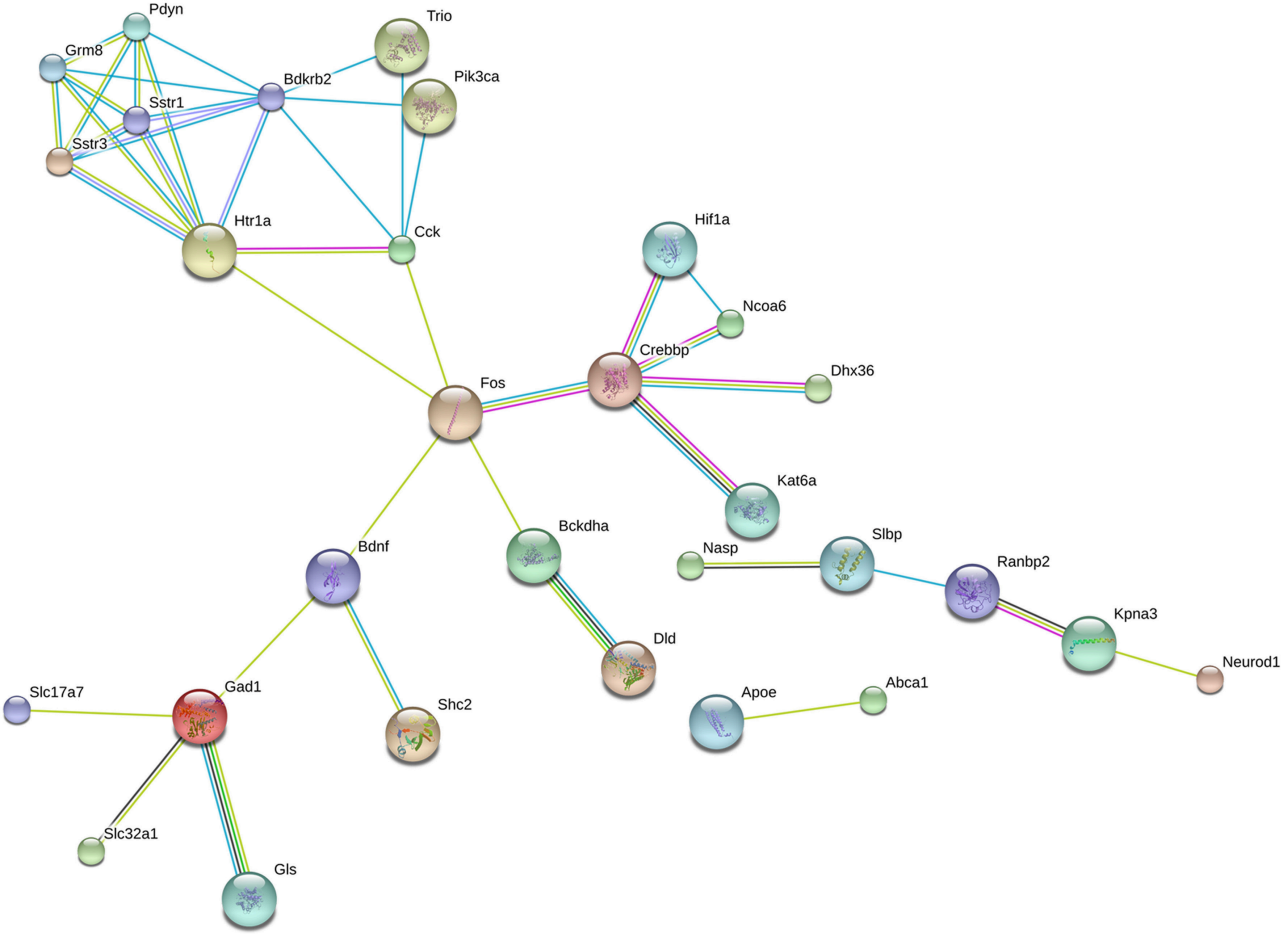
**Predicted interactions among proteins encoded by downregulated genes**. Network composed of proteins whose coding genes were downregulated by long-term DPN treatment. STRING analysis, high confidence value (0.9).

A separate STRING analysis of down-regulated genes was performed to take into account of transcriptional suppression (Figure 3). Predicted interactions among proteins encoded by suppressed genes showed Fos as the hub of the network. One of the clusters was composed of proteins that are responsible for neuropeptide (Pdyn, CCK, Sstr1, Sstr3, Bdkrb2) and neurotransmitter (Htr1a, Grm8) signaling mechanisms. Another cluster was related to neurotransmitter synthesis (Gad1, Gls) and vesicular transport (Slc17a7, Slc32a1). Other clusters serve the initiation of transcription via Fos.

## Discussion

Exploration of selective ERβ agonists has been pursued by the academia and the pharmaceutical industry (Sun et al., 2003; Liu et al., 2008; Clark et al., 2012) since the discovery of the second ER (Kuiper et al., 1996). Molecular, morphological and behavioral studies demonstrated beneficial impact of ERβ agonists on hippocampal functions including anxiety, learning and memory, but the underlying molecular mechanisms remain poorly understood. To explore the impact of ERβ agonists on hippocampal gene expression, we characterized DPN-evoked transcriptional changes in OVX, middle-aged female rats. We found that in this menopausal model, long-term DPN treatment alters hippocampal gene expression, activates several growth/troph factors, modifies transcription and translation, modulates neurotransmitter and neuropeptide signaling mechanisms and exerts neuroprotective effects.

### Previous expression profiling studies in the rodent hippocampus

Estrogenic regulation of the hippocampal transcriptome has already been investigated in mice (Han et al., 2013). Comparison of basal and E2-induced gene expression profiles in wild-type and ERKO mice has clarified the role of ERβ. ERβ exerts a regulatory role on ERα-mediated gene expression and can substitute ERα in the presence of higher levels of E2. The list of the top 30 genes regulated by E2 in the hippocampus of middle-aged ERαKO mice has been published (Han et al., 2013). Importantly, we found 12 overlapping top ERβ-regulated genes in the mouse and rat hippocampus including *Ttr, Otx2, F5, Sostdc1, Folr1, Cldn2, Kl, Slc13a4, Enpp2, Ace, Kcne2*, and *Igf2*.

We have previously examined the effect of treatments with various ER agonists on the innate immune system of the hippocampus in the same model (Sarvari et al., 2014). The study revealed that DPN exerts many overlapping effects with E2 and ERα agonist 16α-estradiol lactone, but there are clear differences. In addition, it supports our previous finding that estrogens regulate neuroinflammatory genes via ERα and ERβ (Sarvari et al., 2011). In another study, we have explored the impact of E2 replacement on the rat hippocampal transcriptome and identified E2 target genes (Sarvari et al., 2015). Comparison with DPN-regulated genes show that 38% of the top E2 target genes also respond to chronic DPN treatment. Overlapping genes include *Ttr, Kl, Cldn2, Prlr, Sostdc1, F5, Igf2, Igfbp2, Slc13a4, Folr1, Htr2c, Col8a2, Mfrp, Otx2, Ace, Mdk, Enpp2, Slco1a5, Ptgds, Kcne2, Scgb1c1, A2m, Aqp1*. This result demonstrates that long-term DPN treatment partly mimics the genomic effects of E2 replacement.

### Chronic DPN administration affects neurotransmitter signaling

#### GABA

GABAergic interneurons control local neuronal circuits in the hippocampus (Freund and Buzsaki, 1996). Six GABA_A_ receptor α subunits (α1–α6) take part in the formation of GABA_A_ complexes. α1, α2, and α4 are found in all sectors of the hippocampus (Wisden et al., 1992). GABA_A_ receptor alpha1 subunit mRNA expression was found in CA1 and CA3 pyramidal neurons and in granule cells (Wisden et al., 1992). Strong α1 immunostaining of GABAergic neurons was also characteristic. All parvalbumin (PV) and half of calretinin interneurons express α1, but calbindin, cholecystokinin (CCK) and VIP interneurons do not (Gao and Fritschy, 1994). We found that long-term ERβ agonist treatment upregulates *Gabra1* which encodes the α1 receptor subunit. Gephyrin, a scaffold protein anchors GABA_A_ receptors to postsynaptic cytoskeleton. The E domain of gephyrin directly binds intracellular residues of 360–374 of α1 (Mukherjee et al., 2011). DPN also upregulates *Gphn*.

Most GABAergic neurons utilize both isoforms of glutamic acid decarboxylase (GAD) in the hippocampal formation (Houser and Esclapez, 1994). GAD67 (GAD1) is spread evenly in the cell, but GAD65 is localized to axon terminals indicating that the former isoform produces GABA for basic cellular processes, the latter for neurotransmission. Estrogenic regulation of the expression of GAD isoforms at mRNA and protein levels has been published (Nakamura et al., 2004). We showed downregulation of *Gad1* after chronic DPN treatment. At axon terminals of inhibitory neurons, vesicular inhibitory amino acid transporters (VIAAT) fill synaptic vesicles with GABA. The transporter protein is highly expressed in the pyramidal and granular layers of the hippocampus (Chaudhry et al., 1998). VIAAT is coded by *Slc32a1*, which was downregulated by DPN. These alterations predict a decrease in the inhibitory tone within the hippocampal formation after long-term DPN treatment.

#### Glutamate

Class I metabotropic glutamate receptors show a distinct pattern of expression in the hippocampus. Metabotropic glutamate receptor 1 (mGluR1) is mainly expressed in granule cells and CA3 pyramidal neurons while mGluR5 is highly expressed in all subfields of the rat hippocampus (Fotuhi et al., 1994). We demonstrated that chronic DPN treatment enhances transcription of *Grm1* and *Grm5*. Estrogenic regulation of AMPA receptors at protein level has already been described in the rat hippocampus (Waters et al., 2009). We found that DPN increases mRNA expression of *Gria2* and *Gria3* coding for GluR2 and GluR3 subunits of the AMPA receptor, respectively.

Glutaminase converts glutamine to glutamate in glutamatergic neurons. Glutaminase IR is found in cell bodies of principal neurons and sparsely, in axon terminals in the rat hippocampus (Altschuler et al., 1985). Glutaminase is also localized to astrocytes (Cardona et al., 2015). We observed that DPN decreases glutaminase mRNA expression. From the three known vesicular glutamate transporters (vGLUT1-3), vGLUT1 is the main subtype expressed in the hippocampus (Fremeau et al., 2004). It packs glutamate into synaptic vesicles of the glutamatergic axon terminals. DPN significantly inhibits transcription of *Slc17a7* which codes for vGLUT1. These findings indicate that long-term DPN treatment decreases the release pool of glutamate in the hippocampus.

#### Serotonin

The HT_1A_ receptor is abundantly expressed in all CA sectors and the dentate gyrus of rat hippocampus (Wright et al., 1995). DPN modestly downregulates *Htr1a*. The two 5-HT_2_ receptors, HT_2A_ and HT_2C_ show distinct expression patterns in the rat hippocampus (Pompeiano et al., 1994). HT_2A_ is expressed in the CA3 sector, HT_2C_ is expressed in the pyramidal cell layer of CA3, the stratum oriens of CA1 and CA2. We showed that both receptor genes (*Htr2a, Htr2c*) are activated in response to DPN. Activation of HT_2A_ receptors has been shown to decrease the expression of BDNF in the granule cell layer without affecting the CA subfields (Vaidya et al., 1997). HT_2A_ receptor is in co-localization with NR1 and GluR2 glutamate receptors residing in dendrites of dentate gyrus neurons. To a lesser extent, the receptor is also expressed in GABAergic interneurons of the dentate gyrus.

#### Acetylcholine

Muscarinic acetylcholine receptors are G protein-coupled receptors. M_1_ mediates primarily the effect of acetylcholine on cognition, plasticity and neuronal excitability (Wei et al., 1994). It has been suggested that expression of muscarinic receptor subtypes depends on serum E2 levels (Cardoso et al., 2010). In concert of this, we found that *Chrm1* and *Chrm5*, which encode M_1_ and M_5_, respectively, are upregulated by DPN. M_5_ is expressed in the hippocampus, especially in pyramidal cells of CA1 (Vilaro et al., 1990).

#### Catecholamines

β1-adrenoceptors are expressed in the brain including CA1, CA3, and dentate gyrus of the hippocampus (Vizi and Kiss, 1998; Paschalis et al., 2009). Beta 1 adrenergic receptor (Adrb1) expression has been described in somatostatin and PV interneurons of the CA1 and CA3 regions (Cox et al., 2008). Long-term DPN treatment slightly downregulates *Adrb1*.

### Modulation of peptidergic signaling

#### Cholecystokinin

The gut peptide CCK is also synthesized in the brain (Innis et al., 1979). The rat hippocampus contains a great number of CCK neurons predominantly located in CA1-2 regions and dentate gyrus (Savasta et al., 1988). CCK-containing terminals are located mainly in the CA1 and dentate gyrus (Handelmann et al., 1981). CCK interneurons represent 10% of GABAergic cells in the hippocampus (Kosaka et al., 1985). We found that DPN suppresses mRNA expression of *Cck*. The finding is in agreement with the decreased GABAergic tone seen after chronic DPN administration.

#### Prodynorphin

Prodynorphin is a precursor of multiple opioid peptides including dynorphin A. After proprotein convertase cleavage, released peptides are stored in large dense-core vesicles in synaptic terminals. Dynorphins, localized primarily to dental granule cells (McGinty et al., 1983), modulate mossy fiber signaling via presynaptic receptors (Weisskopf et al., 1993). We showed that DPN downregulates *Pdyn* mRNA expression.

#### Angiotensin II

Angiotensin II is produced by angiotensin converting enzyme (ACE) from angiotensin I. The rat hippocampus contains high level of angiotensin II (Sirett et al., 1981). Angiotensin II excites CA1 pyramidal cells by disinhibition (Haas et al., 1980). Upregulation of *Ace* is likely to increase the level of angiotensin II after DPN treatment in the hippocampus.

#### Somatostatin receptors

Somatostatin receptor (SSTR) subtypes show wide, distinct but partially overlapping expression in the rat brain including the hippocampus (Kong et al., 1994; Senaris et al., 1994). Somatostatin controls excitatory neurotransmission in the hippocampus via SSTR1 (Cammalleri et al., 2009). SSTR3 mRNA expression has also been reported in the hippocampus (Kong et al., 1994). The receptor protein is associated with cilia of neurons located in the dentate gyrus and CA sectors (Handel et al., 1999). Both *Sstr1* and *Sstr3* are downregulated by DPN.

#### μ-opioid receptor

Opioids primarily target interneurons in the hippocampal formation (Zieglgansberger et al., 1979). Accordingly, μ-opioid receptor IR is localized to multipolar cells in the granular and pyramidal cell layers. High μ-receptor mRNA expression was observed exclusively in GABAergic interneurons (Stumm et al., 2004), and only those that innervate pyramidal cell bodies express μ-opioid receptors (Svoboda et al., 1999). Estrogenic regulation of the receptor has been suggested (Martini et al., 1989). We found that DPN modestly activated transcription of *Oprm1*.

#### Prolactin receptor

Prolactin receptor (PRL-R) is expressed throughout the rat brain (Bakowska and Morrell, 1997; Pi and Grattan, 1998), but the amount of PRL-R mRNA is low in the cerebral cortex (Nagano and Kelly, 1994). We observed robust activation of *Prlr* in the hippocampus after DPN treatment. Prolactin exerts protective effects on the hippocampus against excitotoxicity (Morales et al., 2014) and increases the number of precursor cells (Walker et al., 2012). In PRL-R KO mice, there was an 80% decrease in the number of hippocampus-derived neurospheres and the animals showed learning and memory deficits (Walker et al., 2012). Prolactin has been shown to prevent the decrease of neurogenesis evoked by chronic stress (Torner et al., 2009). Of note, prolactin is synthesized in the rat hippocampus independently from the anterior pituitary (Emanuele et al., 1992).

#### Gonadotropin releasing hormone receptor

Gonadotropin releasing hormone (GnRH) receptor is expressed in the rat hippocampus (Badr and Pelletier, 1987). GnRH receptors are localized to pyramidal cells of CA1, CA3, and granule cells of the dentate gyrus, respectively (Jennes et al., 1995). Expression of GnRH receptor varies during the estrous cycle, with highest expression in proestrous (Savoy-Moore et al., 1980). We found increased mRNA expression of *Gnrhr* after DPN administration. GnRH administration has been reported to change the electrophysiological properties of CA1 neurons (Wong et al., 1990) and regulate spine density in the hippocampus (Prange-Kiel et al., 2008).

#### Galanin receptors

Two galanin receptor (GalR) subtypes mediate the protective and trophic effects of galanin in the hippocampus. GalR1 and GalR2 are predominantly expressed in the CA1 subfield and the dentate gyrus, respectively (Gustafson et al., 1996; Depczynski et al., 1998). The hippocampus receives galaninergic innervations from the septum-basal forebrain complex (Melander et al., 1985). DPN downregulates *Galr2* in the hippocampus. Galanin receptor 2 interacts with neuropeptide Y Y1 receptor in the dentate gyrus and contributes to an antidepressant effect (Narvaez et al., 2015).

#### Corticotropin-releasing hormone receptor 2

Corticotropin-releasing hormone (CRH) receptors are found in the rat hippocampus (Van Pett et al., 2000). The robust downregulation of its expression has recently been reported in rat hippocampus under chronic stress paradigm (Li et al., 2013). We found that DPN enhances mRNA expression of *Crhr2*.

#### PACAP receptor 1

PAC_1_, one of the three receptors for adenylate cyclase activating polypeptide (PACAP), is expressed in the hippocampus (Zhou et al., 2000). PACAP regulates phosphorylation of AMPAR primarily via PAC_1_, which in turn modulates AMPAR function and synaptic plasticity (Toda and Huganir, 2015). PACAP shows neuroprotective activity in various experimental models (Reglodi et al., 2000). PACAP and PAC_1_ are also involved in the regulation of neurodevelopment (Vaudry et al., 1999). PAC_1_ is expressed in a sex-specific manner during development (Shneider et al., 2010). DPN modestly activates transcription of *Adcyap1r1* coding for PAC_1_.

#### Bradykinin receptor 2

B2 bradykinin receptor is widely expressed in neurons of the rat brain including the hippocampal formation (Chen et al., 2000). Bradykinin, released after tissue injury, specifically activates the B2 receptor (Albert-Weissenberger et al., 2013). The receptor increases the excitability of the hippocampus and its susceptibility to seizure (Rodi et al., 2013). We showed downregulation of *Bdkrb2*.

### Control of potassium channels

Voltage-gated potassium channels play an indispensable role in the transition from depolarized to resting state of neurons. For potassium channel coding genes refer to: www.genenames.org/genefamilies/KCN. DPN treatment modulates mRNA expression of genes coding for various voltage-gated potassium channel alfa subunits and a single beta subunit. Alfa subunits include delayed rectifier *Kcna6* (Kv1.6) and *Kcnc2* (Kv3.2), and A-type *Kcnd3* (Kv4.3) family members. The single modulatory beta subunit is *Kcne2*. DPN strongly activates transcription of *Kcne2* which is in line with the presence of estrogen response elements in the promoter region of the gene (Kundu et al., 2008). DPN modestly regulates Ca-activated potassium channel (*Kcnma1*), inwardly rectifying channel (*Kcnj3*), and tetramerization domain-containing 1 (*Kctd1*). With the exception of *Kcnk2* and *Kctd4*, the DPN-controlled potassium channel coding genes were upregulated. Increased expression of *Kcne2* may result in modulation of neuronal excitability via KCNQ2-KCNQ3 channel function in the hippocampus (Tinel et al., 2000).

### Effects on growth/troph hormone signaling

#### Insulin-like growth factor system

IGF2 is abundantly expressed in the rat hippocampus compared to peripheral organs (Ye et al., 2015). IGF2 is a potent regulator of adult hippocampal neurogenesis (Bracko et al., 2012) and memory consolidation (Chen et al., 2011). We found that DPN robustly activated mRNA expression of *Igf2*. DPN also enhanced mRNA expression of *Igfbp2* and *Igf1r*, which are involved in the transport and recognition of IGF2, respectively. IGF1R mediates the effects of serum IGF1 on modulation of synaptic plasticity and hippocampal neurogenesis (Llorens-Martin et al., 2009). Disruption of *Igf1* heavily influences brain development and results in loss of hippocampal granule cells (Beck et al., 1995).

#### Brain derived neurotrophic factor

Brain derived neurotrophic factor (BDNF) mRNA is localized in neurons of the pyramidal cell layer, the granular layer and the hilus of the dentate gyrus (Wetmore et al., 1990). BDNF signaling has a pivotal role in hippocampal neuroprotection, neurogenesis, and affects depression-associated behaviors (Taliaz et al., 2010). BDNF mRNA expression in the hippocampus fluctuates across the estrous cycle and increases in response to E2 (Gibbs, 1998). DPN moderately decreases mRNA expression of *Bdnf*.

#### Fibroblast growth factor

*Fgf1* encodes acidic fibroblast growth factor which is synthesized by the brain vasculature and can induce proliferation of progenitor cells. FGF1 mRNA is also found in the pyramidal cell layer and dentate granule cells of the rat hippocampus (Wilcox and Unnerstall, 1991), although the number of FGF1-IR neurons is rather low in the hippocampus (Stock et al., 1992). We found that DPN moderately activates transcription of *Fgf1*.

#### Midkine

Midkine is a heparin-binding growth factor which is widely expressed in the developing nervous system. It is expressed in NSPCs and enhances their growth and survival (Zou et al., 2006). The expression of midkine is limited to a few cell types in the adult brain (Bloch et al., 1992). DPN moderately upregulates *Mdk* expression.

### Increase of klotho signaling

Klotho is a pleiotropic gene the overexpression of which extends lifespan in mice (Kurosu et al., 2005). Klotho is highly expressed in the choroid plexus and in neurons of the hippocampus (Kuro-o et al., 1997). It encodes a type I membrane protein which can be cleaved by metalloproteases and released from the cell membrane. Klotho promotes FGF-23 signaling, inhibits insulin/IGF1 signaling and regulates Ca^++^ homeostasis (Imura et al., 2007). In mice, systemic overexpression of klotho improves learning and memory, and elevates synaptic NR2B in a subunit-specific manner through posttranscriptional mechanism (Dubal et al., 2014). We demonstrated that DPN robustly activates mRNA expression of klotho.

### Regulation of transcription

The homeobox-containing transcription factor Otx2 plays an indispensable role in normal brain development, but its role is enigmatic in adulthood. The passage of Otx2 between cells by non-conventional mechanism gives another layer of complexity to the mechanism of action of Otx2 (Prochiantz and Theodore, 1995). Accumulation of Otx2 is required for the maturation of PV-containing interneurons (Sugiyama et al., 2008). Long-term DPN treatment highly activates mRNA expression of Otx2 which may influence hippocampal plasticity. DPN also increases mRNA expression of serum response factor (Srf), myelin transcription factor 1 (Myt1), muscle segment homeobox (Msx1), pre-B cell leukemia homeobox (Pbx1), transcription factor 12 (Tcf12), and mediator 1 (Med1). Srf controls activity-dependent immediate-early gene expression and synaptic strength in CA1 pyramidal neurons (Ramanan et al., 2005). Mice with deletion of *Srf* in the forebrain show impaired LTD and selective deficit in explicit spatial memory (Etkin et al., 2006). These findings suggest a crucial role for Srf in learning. Myt1 is expressed in neural progenitors and oligodendrocytes, and is likely to regulate oligodendrocyte differentiation (Armstrong et al., 1995). In the hippocampus, Msx1 is localized to astrocytes, oligodendrocytes, and immature oligodendrocytes (Ramos et al., 2004). Pbx1 is highly expressed in the developing rat brain and its expression is maintained in TuJ1-IR postmitotic neurons (Redmond et al., 1996). We showed that DPN decreases mRNA expression of *Fos*, which is in line with the presence of an estrogen response element-like sequence in its promoter region (Cattaneo and Maggi, 1990).

Cyclin-dependent kinases (Cdk) form complexes with cyclins and operate as transcription factors. Depletion of *Cdk10* impairs progenitor cell survival (Yeh et al., 2013), therefore DPN-evoked upregulation of *Cdk10* may improve neural stem and progenitor cell (NSPC) survival. Deletion of *Cdkn1c* initially causes increased neurogenesis, while on the long run leads to impaired neurogenesis in the hippocampus (Furutachi et al., 2013). We found that DPN enhances *Cdkn1c* mRNA expression. DPN inversely modulates the transcription of coregulators, activates *Ncor1* and suppresses *Crebbp* and *Ncoa6*, among others. DPN also activates the transcription of *Polr2b* encoding DNA-directed RNA polymerase II polypeptide B. DPN can affect mRNA splicing and metabolism by upregulating splicing factors (Sfrs11, Srrm1), spliceosome components (Snrpg) and ribonucleoproteins (Hnrnpd, Hnrnpf, Prpf3). These findings suggest that DPN widely alters the transcriptional machinery in the hippocampus.

### Functional considerations

#### Neuronal plasticity

In females, structure and function of the tri-synaptic circuit of the hippocampal formation is responsive to changes of gonadal hormone levels. The neuronal response to E2, which takes place within 15 min, includes dendritic remodeling and synaptic turnover. The role of fast, nongenomic effects has been explored and recognized, while the role of genomic effects has remained unexplored. This study demonstrates that selective activation of ERβ leads to robust alteration of mRNA expression of growth/troph factor genes and modest changes of neurotransmitter and peptide receptor genes.

In the hippocampus, AMPA-type glutamate receptors (AMPAR) are composed of GluR1/2 and GluR2/3 heterodimers. As AMPAR mediates the majority of fast synaptic transmission, the modulation of its composition and phosphorylation control synaptic strength and cell firing (Huganir and Nicoll, 2013). Upregulation of genes coding for AMPA receptor subunits GluR2 (*Gria2*) and GluR3 (*Gria3*) may increase constitutive insertion of GluR2/3 heterodimers shifting the composition of synaptic AMPA receptors. The DPN-evoked increase of GluR2 content may reduce Ca^++^ influx and decrease the potential of excitatory events (Hume et al., 1991). The level and site of GluR1 phosphorylation are regulated by various receptors including β-adrenergic receptor, m1 acetylcholine receptor (Seol et al., 2007) and PAC_1_ (Toda and Huganir, 2015), among others. Alterations in the expression of these receptors may affect phosphorylation state of GluR1. In addition, downregulation of vesicular glutamate transporter 1 (Slc17a7) suggests a decreased synaptic glutamate pool in pyramidal and dentate granule cells. We also showed significant suppression of vesicular GABA transporter (Slc32a1) expression which indicates a decreased inhibitory tone in the hippocampus after chronic DPN treatment. Decreased *Cck* expression implicates CCK interneurons in the alterations related to inhibitory neurotransmission.

#### Neurogenesis

Neuronal plasticity also includes replacement of nonfunctional cells by the proliferation, differentiation and migration of NSPCs in the adult hippocampus. Proliferating (Ki-67+) and migrating (DC+) cells of the dentate gyrus express ERβ and ERα mRNA (Isgor and Watson, 2005). Extranuclear ERβ is localized to DC-containing cells in the dentate gyrus of adult rats (Herrick et al., 2006). Various laboratories have demonstrated that selective ERβ agonists promote neurogenesis in the dentate gyrus (Mazzucco et al., 2006; Clark et al., 2012). Our study identified robust, ERβ-dependent transcriptional regulation of key genes that are involved in NSPC proliferation and survival. Important upregulated genes associated with neurogenesis were *Igf2* (along with *Igfbp2, Igf1r*), *Fgf1, Mdk, Prlr, Cdk10*, and *Cdkn1c*. Insulin-like growth factor 2 is the master regulator of adult neurogenesis in the subgranular zone (Bracko et al., 2012; Ouchi et al., 2013). In accord, Igf2 is expressed in progenitor cells and can regulate cell proliferation and differentiation in autocrine manner (Ferron et al., 2015). Acidic fibroblast growth factor maintains proliferation, self-renewal and survival of NSPC via binding to FGF receptors (Ray and Gage, 2006; Ma et al., 2009). Midkine enhances the growth and survival of NSPC (Zou et al., 2006). Prolactin can induce adult neurogenesis in the hippocampus (Shingo et al., 2003; Larsen and Grattan, 2012). Regulation of cyclin-dependent kinase activity affects proliferation and survival of progenitor cells (Furutachi et al., 2013; Yeh et al., 2013). Of note, downregulation of *Bdnf* may also have a strong impact on neurogenesis (Maisonpierre et al., 1990). These results demonstrate that the ERβ agonist DPN modulates the transcription of several major regulators of NSPC proliferation, differentiation, and survival.

#### Neuroprotection

Both ERα and ERβ selective agonists exert neuroprotective effects (Zhao et al., 2004). It is likely that multiple mechanisms are responsible for the neuroprotective action of ERβ agonists, and one of the putative mechanisms is myelination. In a mouse experimental autoimmune encephalomyelitis model, DPN promotes myelination and protects neurons (Khalaj et al., 2013). Cellular targets of the ERβ agonist in this case are cells of the oligodendrocyte lineage. DPN can enhance oligodendrocyte maturation via klotho signaling (Chen et al., 2013) and via transcription factors such as Msx1 and Myt1 (Armstrong et al., 1995). Upregulation of *Mbp* supports this notion.

DPN robustly activates transcription of klotho. Klotho expression is detectable in the rat brain including the hippocampus (Clinton et al., 2013). It protects neurons against neurodegeneration and oxidative stress via regulation of members of the redox system (Zeldich et al., 2014). In a mouse model, inverse correlation was shown between klotho expression and Alzheimer's disease phenotype (Kuang et al., 2014). Neurohormones, such as prolactin and PACAP protect hippocampal neurons following excitotoxic insult (Morales et al., 2014) and ischemia (Ohtaki et al., 2006), respectively. Upregulation of their receptors may potentiate the protective effects of these neurohormones in the hippocampus.

Another protective mechanism is mediated by transient receptor potential melastatin 7 (Trpm7) which is downregulated by DPN. Trpm7 in hippocampal neurons detects extracellular divalent cations (Wei et al., 2007). Inhibition of Trpm7 expression blocks Trpm7 currents, anoxic Ca^++^-uptake, ROS production and anoxic death (Aarts et al., 2003). Suppression of Trpm7 makes neurons resistant to ischemic death following brain ischemia (Sun et al., 2009). These data suggest that downregulation of *Trpm7* provides protection against ischemia after long-term DPN treatment.

It has been demonstrated that some E2 binding sites were associated with mitochondria in dendritic shafts and spines of the rat hippocampus (Milner et al., 2008). In accord, E2 modulates the brain mitochondrial proteome (Nilsen et al., 2007) and regulates oxidative metabolism in mitochondria (Irwin et al., 2008). DPN significantly increases the association of ERβ with mitochondria and enhances protein expression of ATP synthase in the hippocampus (Irwin et al., 2012). We found that DPN modestly enhances mRNA expression of ATP synthase (*Atp5l, Atp5e*), cytochrome c oxidase VIII (*Cox8b*) and some mitochondrial ribosomal protein genes (*Mrpl42, Mrps16, Mrps27*) which may reflect elevated mitochondrial ATP synthesis supporting hippocampal functions.

Glutathione peroxidase is the major enzyme to remove the excess of cytosolic and mitochondrial hydrogen peroxide in neurons (Desagher et al., 1996). Moderate upregulation of *Gpx1* may provide enhanced protection against hydroxyl radical formation in the hippocampus.

Modulation of innate immune processes in the brain may also affect neuroprotection. We have recently published that DPN attenuates mRNA expression of some macrophage-associated and complement genes in the hippocampus (Sarvari et al., 2014). Here we showed upregulation of additional immune genes such as *Cd59, Irak1, Cxcr4*, and *Mif*. Upregulation of *Cd59* and *Irak1* may enhance the protection against homologous complement lysis (Singhrao et al., 2000) and promote the initial response to TLR stimulation (Kawagoe et al., 2008), respectively.

Concerning limitations, first we note that pharmacological approaches in ERβ research are hindered by the moderate selectivity of ERβ agonists. In a chronic paradigm it seems reasonable to assume that nonselective actions on ERα may occur. However, the lack of the hallmark of ERα, namely its proliferating effect in the uterus, does not support this notion. In the hippocampus, DPN in part exerts overlapping transcriptional effects with E2, but there are clear differences (Sarvari et al., 2015). In addition, DPN distinctly regulates mRNA expression of some immune genes (*Mrc1, Cd163, Cd200r1, Cfb, Cfp*) in the hippocampus (Sarvari et al., 2014). These data support the view that at the applied dose DPN selectively activates ERβ. Second, during the course of treatment DPN modulates the expression of growth factors, transcriptional regulators and transcription factors, among others. Therefore, primary and secondary effects on gene transcription can be hardly identified at the end of treatment. Third, one of the most responsive genes is *Ttr*. It is in accord with the presence of a putative estrogen-response element within the 5′ flanking region of *TTR* (Martinho et al., 2013). Transthyretin is mainly synthesized in the brain by the choroid plexus, and its expression is regulated by E2 (Tang et al., 2004). Although mRNA expression of *Ttr* in the hippocampus (Wang et al., 2014) and increased level of the protein in the dentate gyrus during memory consolidation (Monopoli et al., 2011) support local synthesis, contribution of the choroid plexus can't be ruled out since we can't exclude the presence of traces of choroid plexus in the dissected hippocampus. Fourth, the transcriptional effects of ER ligands partly depend on the chemical structure of the compound (Kuiper et al., 1998). Therefore, it is likely that various agonists exert slightly different transcriptional effects, i.e., some genes may show DPN specific regulation.

**Figure 4:**
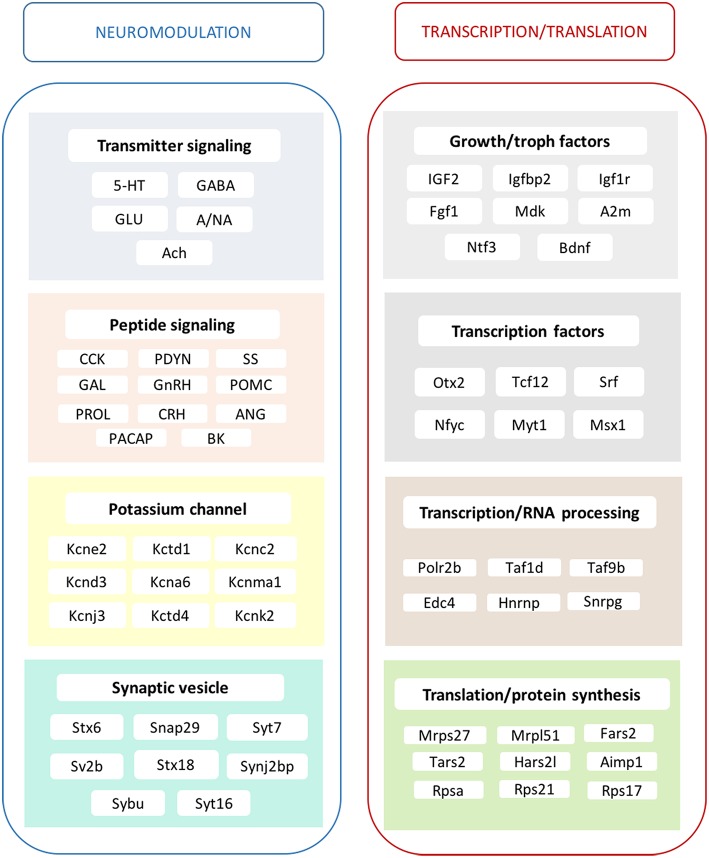
**Main effects of ERβ agonist DPN on the hippocampal transcriptome**. The alterations in transmitter signaling include the 5-HT, serotonergic; GABA, GABA-ergic; GLU, glutamatergic; A/NA, adrenergic/noradrenergic; and Ach, cholinergic systems. In the case of peptidergic signaling mechanisms, the local production of CCK, cholecystokinin and PDYN, prodynorphin is influenced. Various peptide hormone receptors are also regulated by DPN. This group reflects changes in GAL, galanin; GnRH, gonadotropin-releasing hormone; POMC, pro-opiomelanocortin; PRL, prolactin; CRH, corticotropin-releasing hormone; ANG, angiotensin; PACAP, pituitary adenylate cyclase-activating polypeptide; and BK, bradykinin neurotransmission/modulation. Growth hormone and trophic factor production is also highly targeted. A strong regulatory effect is exerted upon certain potassium channels. Synaptic vesicle proteins are also regulated. Transcription factors show altered expression accompanied with modification of RNA processing, translation and protein synthesis. These complex mechanisms may regulate neurogenesis and synaptic plasticity and elucidate the molecular background of hippocampus-related physiological processes and symptoms observed after DPN administration, as summarized partly in Supplementary Table 4.

Summing up, long-term ERβ agonist treatment evokes a broad array of changes in the hippocampal transcriptome of middle-aged, ovariectomized rats (Figure 4). A significant set of DPN-regulated genes was reminiscent to those seen in response to E2 (Sarvari et al., 2015). The identified basic cellular and neuronal network mechanisms are utilized in the regulation of neuronal plasticity, neurogenesis, and neuroprotection in the hippocampal formation. These events are known to influence learning, memory, spatial navigation and mood/behavior-related events (Supplementary Table 4). Based on these results, we propose that ERβ agonist treatment may represent a safe and effective estrogen replacement therapy to preserve the integrity of the hippocampal formation in the postmenopausal period.

## Author contributions

ZL, MS conceived and designed experiments. MS, IK, EH, AR performed experiments. MS, NS, ZL analyzed data. MS, ZL wrote the paper.

## Funding

This work was supported by the Hungarian Scientific Research Fund Grant OTKA K100722 for ZL.

### Conflict of Interest Statement

The authors declare that the research was conducted in the absence of any commercial or financial relationships that could be construed as a potential conflict of interest.
