# Supplementary material for: Long-Term Estrogen Receptor Beta Agonist Treatment Modifies the Hippocampal Transcriptome in Middle-Aged Ovariectomized Rats
Source: Front Cell Neurosci. 2016 Jun 10;10:149. doi: 10.3389/fncel.2016.00149 (PMC4901073; doi:10.3389/fncel.2016.00149)
Supplement: Supplementary file 1 [file Presentation1.PDF]

SUPPLEMENTARY FIGURES AND TABLES

Supplementary Table 1. List of abbreviations in alphabetical order

Symbol	Description
A2m	alpha-2-macroglobulin
Abca1	ATP-binding cassette, subfamily A (ABC1), member 1
Abcc1	ATP-binding cassette, subfamily C (CFTR/MRP), member 1
Abcd2	ATP-binding cassette, subfamily D (ALD), member 2
Abhd8	abhydrolase domain containing 8
Acadvl	acyl-CoA dehydrogenase, very long chain
Acbd4	acyl-CoA binding domain containing 4
Ace	angiotensin I converting enzyme (peptidyl-dipeptidase A) 1
Actr2	ARP2 actin-related protein 2 homolog (yeast)
Acyp2	acylphosphatase 2, muscle type
Adam11	ADAM metallopeptidase domain 11
Adcyap1r1	adenylate cyclase activating polypeptide 1 receptor 1
Agpat3	1-acylglycerol-3-phosphate O-acyltransferase 3
Agtpbp1	ATP/GTP binding protein 1
Aig1	androgen-induced 1
Aimp1	aminoacyl tRNA synthetase complex-interacting multifunctional protein 1
Akap2	A kinase (PRKA) anchor protein 2
Akip1	A kinase (PRKA) interacting protein 1
Akt1s1	AKT1 substrate 1 (proline-rich)
Alkbh	alkB, alkylation repair homolog (E. coli)
Amigo2	adhesion molecule with Ig like domain 2
Amn1	antagonist of mitotic exit network 1 homolog (S. cerevisiae)
Amt	aminomethyltransferase
Anapc11	anaphase promoting complex subunit 11
Ank3	ankyrin 3, node of Ranvier
Apc2	adenomatosis polyposis coli 2
Aqp1	aquaporin 1
Arfgap1	ADP-ribosylation factor GTPase activating protein 1
Arhgap32	Rho GTPase activating protein 32
Arhgef26	Rho guanine nucleotide exchange factor (GEF) 26
Arl10	ADP-ribosylation factor-like 10
Arl13b	ADP-ribosylation factor-like 13B
Arl6ip4	ADP-ribosylation-like factor 6 interacting protein 4
Arpp19	cAMP-regulated phosphoprotein 19
Asap1	ArfGAP with SH3 domain, ankyrin repeat and PH domain 1
Ash1l	ash1 (absent, small, or homeotic)-like (Drosophila)
Ate1	arginyltransferase 1
Atox1	ATX1 antioxidant protein 1 homolog (yeast)
Atp2a2	ATPase, Ca++ transporting, cardiac muscle, slow twitch 2
Atp5e	ATP synthase, H+ transporting, mitochondrial F1 complex, epsilon subunit
Atp5l	ATP synthase, H+ transporting, mitochondrial Fo complex, subunit G

B9d1	B9 protein domain 1
Baz2b	bromodomain adjacent to zinc finger domain, 2B
Bckdha	branched chain ketoacid dehydrogenase E1, alpha polypeptide
Bcl2l1	Bcl2-like 1
Bdkrb2	bradykinin receptor B2
Bloc1s1	biogenesis of lysosomal organelles complex-1, subunit 1
C2cd2l	C2 calcium-dependent domain containing 2-like
Cacna1a	calcium channel, voltage-dependent, P/Q type, alpha 1A subunit
Calm3	calmodulin 3
Calml4	calmodulin-like 4
Camsap2	calmodulin regulated spectrin-associated protein family, member 2
Cbfa2t3	core-binding factor, runt domain, alpha subunit 2; translocated to, 3
Ccar1	cell division cycle and apoptosis regulator 1
Ccdc25	coiled-coil domain containing 25
Cd59	CD59 molecule, complement regulatory protein
Cdk10	cyclin-dependent kinase 10
Cdk13	cyclin-dependent kinase 13
Cdk7	cyclin-dependent kinase 7
Cdkn2c	cyclin-dependent kinase inhibitor 2C (p18, inhibits CDK4)
Cdv3	carnitine deficiency-associated gene expressed in ventricle 3 homolog (mouse)
Cerk	ceramide kinase
Ces5a	carboxylesterase 5A
Chd2	chromodomain helicase DNA binding protein 2
Chrm1	cholinergic receptor, muscarinic 1
Chrm4	cholinergic receptor, muscarinic 4
Chst11	carbohydrate (chondroitin 4) sulfotransferase 11
Chst2	carbohydrate sulfotransferase 2
Cldn11	claudin 11
Cldn12	claudin 12
Cldn2	claudin 2
Clptm1l	CLPTM1-like
Cmtm4	CKLF-like MARVEL transmembrane domain containing 4
Cnfn	cornifelin
Cnot6l	CCR4-NOT transcription complex, subunit 6-like
Cntn2	contactin 2 (axonal)
Col8a2	collagen, type VIII, alpha 2
Commd10	COMM domain containing 10
Commd6	COMM domain containing 6
Copg	coatomer protein complex, subunit gamma
Copz1	coatomer protein complex, subunit zeta 1
Cox11	COX11 cytochrome c oxidase assembly homolog (yeast)
Cpox	coproporphyrinogen oxidase
Cpsf3	cleavage and polyadenylation specific factor 3, 73kDa
Crebbp	CREB binding protein
Ctdnep1	CTD nuclear envelope phosphatase 1

Ctdp1	CTD (carboxy-terminal domain, RNA polymerase II, polypeptide A) phosphatase, subunit 1
Ctsh	cathepsin H
Cxcr4	chemokine (C-X-C motif) receptor 4
Csnk2a2	casein kinase 2, alpha prime polypeptide
Csrnp2	cysteine-serine-rich nuclear protein 2
Dag1	dystroglycan 1 (dystrophin-associated glycoprotein 1)
Dcaf6	DDB1 and CUL4 associated factor 6
Dck	deoxycytidine kinase
Dclk2	doublecortin-like kinase 2
Dda1	DET1 and DDB1 associated 1
Ddit3	DNA-damage inducible transcript 3
Ddx26b	DEAD/H (Asp-Glu-Ala-Asp/His) box polypeptide 26B
Ddx27	DEAD (Asp-Glu-Ala-Asp) box polypeptide 27
Ddx3x	DEAD (Asp-Glu-Ala-Asp) box polypeptide 3, X-linked
Ddx49	DEAD (Asp-Glu-Ala-Asp) box polypeptide 49
Ddx58	DEAD (Asp-Glu-Ala-Asp) box polypeptide 58
Ddx6	DEAD (Asp-Glu-Ala-Asp) box polypeptide 6
Dhx36	DEAH (Asp-Glu-Ala-His) box polypeptide 36
Dld	dihydrolipoamide dehydrogenase
Dlgap1	discs, large (Drosophila) homolog-associated protein 1
Dlgap3	discs, large (Drosophila) homolog-associated protein 3
Dnaja3	DnaJ (Hsp40) homolog, subfamily A, member 3
Dnmt3a	DNA (cytosine-5-)-methyltransferase 3 alpha
Dohh	deoxyhypusine hydroxylase/monooxygenase
Dos	downstream of Stk11
Dpp8	dipeptidylpeptidase 8
Dtnbp1	distrobrevin binding protein 1
Edc4	enhancer of mRNA decapping 4
Eif3d	eukaryotic translation initiation factor 3, subunit D
Eif3e	eukaryotic translation initiation factor 3, subunit E
Eif3s6ip	eukaryotic translation initiation factor 3, subunit 6 interacting protein
Eif4g1	eukaryotic translation initiation factor 4 gamma, 1
Eif5a2	eukaryotic translation initiation factor 5A2
Elavl3	ELAV (embryonic lethal, abnormal vision, Drosophila)-like 3 (Hu antigen C)
Elfn2-ps1	extracellular leucine-rich repeat and fibronectin type III domain containing 2, pseudogene 1
Elovl6	ELOVL fatty acid elongase 6
Eml4	echinoderm microtubule associated protein like 4
Enpp2	ectonucleotide pyrophosphatase/phosphodiesterase 2
Ensa	endosulfine alpha
Ep400	E1A binding protein p400
Ephb3	Eph receptor B3
Epn2	epsin 2
Erbb4	v-erb-a erythroblastic leukemia viral oncogene homolog 4 (avian)
F5	coagulation factor V (proaccelerin, labile factor)
Fam134b	family with sequence similarity 134, member B

Fam183b	family with sequence similarity 183, member B
Fam18a	family with sequence similarity 18, member A
Fam195a	family with sequence similarity 195, member A
Fam91a1	family with sequence similarity 91, member A1
Fars2	phenylalanyl-tRNA synthetase 2, mitochondrial
Fbrsl1	fibrosin-like 1
Fbxl6	F-box and leucine-rich repeat protein 6
Fermt2	fermitin family member 2
Fibcd1	fibrinogen C domain containing 1
Fkbp1a	FK506 binding protein 1a
Fkbp2	FK506 binding protein 2
Fmod	fibromodulin
Fnbp1	formin binding protein 1
Fnbp4	formin binding protein 4
Fndc3a	fibronectin type III domain containing 3a
Folr1	folate receptor 1 (adult)
Fos	FBJ osteosarcoma oncogene
Frmd4b	FERM domain containing 4B
Fubp3	far upstream element (FUSE) binding protein 3
Fxyd1	FXYD domain-containing ion transport regulator 1
Gapdh	glyceraldehyde-3-phosphate dehydrogenase
Gas5	growth arrest specific 5
Gca	grancalcin
Gga3	golgi associated, gamma adaptin ear containing, ARF binding protein 3
Gigyf1	GRB10 interacting GYF protein 1
Gigyf2	GRB10 interacting GYF protein 2
Gkap1	G kinase anchoring protein 1
Gls	glutaminase
Gltscr1	glioma tumor suppressor candidate region gene 1
Gnb5	guanine nucleotide binding protein (G protein), beta 5
Gng12	guanine nucleotide binding protein (G protein), gamma 12
Gns	glucosamine (N-acetyl)-6-sulfatase
Gpatch4	G patch domain containing 4
Grk6	G protein-coupled receptor kinase 6
Grm1	glutamate receptor, metabotropic 1
Grm8	glutamate receptor, metabotropic 8
Grxcr1	glutaredoxin, cysteine rich 1
Gtf3c6	general transcription factor IIIC, polypeptide 6, alpha
Gtpbp6	GTP binding protein 6 (putative)
Hapln2	hyaluronan and proteoglycan link protein 2
Hars2l	histidyl-tRNA synthetase 2-like
Hcfc1r1	host cell factor C1 regulator 1 (XPO1-dependent)
Hectd1	HECT domain containing 1
Hif1a	hypoxia-inducible factor 1, alpha subunit (basic helix-loop-helix transcription factor)
Hip1	huntingtin interacting protein 1

Hist2h2aa3	histone cluster 2, H2aa3
Hk1	hexokinase 1
Hnrnpf	heterogeneous nuclear ribonucleoprotein F
Hnrnpu	heterogeneous nuclear ribonucleoprotein U
Hnrpd	heterogeneous nuclear ribonucleoprotein D
Hsp90ab1	heat shock protein 90 alpha (cytosolic), class B member 1
Htr2c	5-hydroxytryptamine (serotonin) receptor 2C
Icam5	intercellular adhesion molecule 5, telencephalin
Ide	insulin degrading enzyme
Igf2	insulin-like growth factor 2
Igfbp2	insulin-like growth factor binding protein 2
Il16	interleukin 16
Immt	inner membrane protein, mitochondrial
Inpp5f	inositol polyphosphate-5-phosphatase F
Ints6	integrator complex subunit 6
Irak1	interleukin-1 receptor-associated kinase 1
Isca1	iron-sulfur cluster assembly 1 homolog (S. cerevisiae)
Itsn2	intersectin 2
Kat6a	K(lysine) acetyltransferase 6A
Kbtbd2	kelch repeat and BTB (POZ) domain containing 2
Kcna6	potassium voltage gated channel, shaker related subfamily, member 6
Kcnc2	potassium voltage gated channel, Shaw-related subfamily, member 2
Kcnd3	potassium voltage-gated channel, Shal-related subfamily, member 3
Kcne2	potassium voltage-gated channel, Isk-related family, member 2
Kcnj3	potassium inwardly-rectifying channel, subfamily J, member 3
Kcnk2	potassium channel, subfamily K, member 2
Kcnma1	potassium large conductance calcium-activated channel, subfamily M, alpha member 1
Kctd1	potassium channel tetramerisation domain containing 1
Kctd4	potassium channel tetramerisation domain containing 4
Kdm6a	lysine (K)-specific demethylase 6A
Khsrp	KH-type splicing regulatory protein
Kif1a	kinesin family member 1A
Kl	Klotho
Klc1	kinesin light chain 1
Klc3	kinesin light chain 3
Klf13	Kruppel-like factor 13
Klf3	Kruppel-like factor 3 (basic)
Kpna3	karyopherin alpha 3
Lace1	lactation elevated 1
Lama3	laminin, alpha 3
Ldb2	LIM domain binding 2
Lgals1	lectin, galactoside-binding, soluble, 1
Lin7c	lin-7 homolog C (C. elegans)
LOC100188932	dolichyl-diphosphooligosaccharide--protein glycosyltransferase subunit 4
LOC100294508	dyslexia susceptibility 2-like

LOC100360606	zinc finger, RAN-binding domain containing 1 protein-like
LOC100361182	armadillo repeat containing, X-linked 5
LOC100362458	rCG23949-like
LOC100363114	eukaryotic translation initiation factor 4 gamma, 3-like
LOC100365089	rCG50929-like
LOC100365679	DENN/MADD domain containing 5B-like
LOC294154	similar to chromosome 6 open reading frame 106 isoform a
LOC301124	hypothetical LOC301124
LOC501091	similar to Discs large homolog 5 (Placenta and prostate DLG) (Discs large protein P-dlg)
LOC680835	similar to cullin 7
LOC681251	hypothetical protein LOC681251
LOC681825	similar to Prefoldin subunit 3 (Von Hippel-Lindau-binding protein 1) (VHL-binding protein 1)
LOC682967	similar to Protein disulfide-isomerase precursor (Thioredoxin domain-containing protein 10)
LOC683519	similar to ribosomal protein, mitochondrial, S22
LOC684258	similar to coiled-coil-helix-coiled-coil-helix domain containing 7
LOC686944	similar to Zinc finger protein 45 (BRC1744)
LOC687029	similar to differentially expressed in B16F10 1
LOC688018	similar to SH3-domain binding protein 3
LOC688549	hypothetical protein LOC688549
LOC688786	similar to CG14483-PA
LOC689298	hypothetical protein LOC689298
LOC690769	similar to zinc ring finger protein 1
LOC691468	similar to Zinc finger protein 84 (Zinc finger protein HPF2)
Lrp11	low density lipoprotein receptor-related protein 11
Lrrc4b	leucine rich repeat containing 4B
Lrsam1	leucine rich repeat and sterile alpha motif containing 1
Lsm7	LSM7 homolog, U6 small nuclear RNA associated (S. cerevisiae)
Maged2	melanoma antigen, family D, 2
Map7	microtubule-associated protein 7
Map7d2	MAP7 domain containing 2
Mapk10	mitogen activated protein kinase 10
Mapk8ip2	mitogen-activated protein kinase 8 interacting protein 2
Mapre1	microtubule-associated protein, RP/EB family, member 1
Mars	methionine-tRNA synthetase
Mast3	microtubule associated serine/threonine kinase 3
Mau2	Mau2 chromatid cohesion factor homolog (C. elegans)
Mbp	myelin basic protein
Mcu	mitochondrial calcium uniporter
Mdk	midkine
Med1	mediator complex subunit 1
Mef2bnb	MEF2B neighbor
Mex3b	mex3 homolog B (C. elegans)
Mfrp	membrane frizzled-related protein
Mga	MAX gene associated
Mgea5	meningioma expressed antigen 5 (hyaluronidase)

Mgll	monoglyceride lipase
Mif	macrophage migration inhibitory factor
Mkl1	megakaryoblastic leukemia (translocation) 1
Mlec	malectin
Mllt6	myeloid/lymphoid or mixed-lineage leukemia (trithorax homolog); translocated to, 6
Mob1a	MOB kinase activator 1A
Mob1b	MOB kinase activator 1B
Mrpl42	mitochondrial ribosomal protein L42
Mrpl51	mitochondrial ribosomal protein L51
Mrps16	mitochondrial ribosomal protein S16
Mrps27	mitochondrial ribosomal protein S27
Mxd4	Max dimerization protein 4
Myt1	myelin transcription factor 1
Nacad	NAC alpha domain containing
Nasp	nuclear autoantigenic sperm protein (histone-binding)
Nat14	N-acetyltransferase 14
Nbr1	neighbor of Brca1 gene 1
Ncln	nicalin
Ncoa6	nuclear receptor coactivator 6
Ncor1	nuclear receptor co-repressor 1
Ndfip2	Nedd4 family interacting protein 2
Ndufa12	NADH dehydrogenase (ubiquinone) 1 alpha subcomplex, 12
Ndufv3	NADH dehydrogenase (ubiquinone) flavoprotein 3
Nedd4	neural precursor cell expressed, developmentally down-regulated 4
Nedd9	neural precursor cell expressed, developmentally down-regulated 9
Nek1	NIMA (never in mitosis gene a)-related kinase 1
Neurod1	neurogenic differentiation 1
Nf2	neurofibromin 2 (merlin)
Nfia	nuclear factor I/A
Nfyc	nuclear transcription factor-Y gamma
Nicn1	nicolin 1
Nip30	NEFA-interacting nuclear protein NIP30
Nol4	nucleolar protein 4
Nolc1	nucleolar and coiled-body phosphoprotein 1
Nova2	neuro-oncological ventral antigen 2
Nucks1	nuclear casein kinase and cyclin-dependent kinase substrate 1
Nudcd3	NudC domain containing 3
Nupl1	nucleoporin like 1
Otx2	orthodenticle homeobox 2
Pabpc1	poly(A) binding protein, cytoplasmic 1
Pabpn1	poly(A) binding protein, nuclear 1
Pacrgl	PARK2 co-regulated-like
Padi2	peptidyl arginine deiminase, type II
Paf1	Paf1, RNA polymerase II associated factor, homolog (S. cerevisiae)
Pank2	pantothenate kinase 2 (Hallervorden-Spatz syndrome)

Papolg	poly(A) polymerase gamma
Pbx1	pre-B-cell leukemia homeobox 1
Pbxip1	pre-B-cell leukemia homeobox interacting protein 1
Pcbd2	pterin 4 alpha carbinolamine dehydratase/dimerization cofactor of hepatocyte nuclear factor 1 alpha 2
Pde4d	phosphodiesterase 4D, cAMP-specific
Pdia4	protein disulfide isomerase family A, member 4
Pdlim5	PDZ and LIM domain 5
Pdrg1	p53 and DNA damage regulated 1
Pds5b	PDS5, regulator of cohesion maintenance, homolog B (S. cerevisiae)
Pdzd4	PDZ domain containing 4
Pes1	pescadillo homolog 1, containing BRCT domain (zebrafish)
Pfkfb2	6-phosphofructo-2-kinase/fructose-2,6-biphosphatase 2
Pfn1	profilin 1
Pgrmc2	progesterone receptor membrane component 2
Pias1	protein inhibitor of activated STAT, 1
Pik3ca	phosphoinositide-3-kinase, catalytic, alpha polypeptide
Pitpnm2	phosphatidylinositol transfer protein, membrane-associated 2
Pla2g6	phospholipase A2, group VI (cytosolic, calcium-independent)
Plekhh1	pleckstrin homology domain containing, family H (with MyTH4 domain) member 1
Plekho1	pleckstrin homology domain containing, family O member 1
Pls3	plastin 3
Pltp	phospholipid transfer protein
Pnisr	PNN-interacting serine/arginine-rich protein
Pnkd	paroxysmal nonkinesiogenic dyskinesia
Pnmal2	PNMA-like 2
Polr2b	polymerase (RNA) II (DNA directed) polypeptide B
Pou3f3	POU class 3 homeobox 3
Ppp1r1a	protein phosphatase 1, regulatory (inhibitor) subunit 1A
Prcp	prolylcarboxypeptidase (angiotensinase C)
Prelid1	PRELI domain containing 1
Prkag2	protein kinase, AMP-activated, gamma 2 non-catalytic subunit
Prlr	prolactin receptor
Prpf3	PRP3 pre-mRNA processing factor 3 homolog (S. cerevisiae)
Prrc2c	proline-rich coiled-coil 2C
Psip1	PC4 and SFRS1 interacting protein 1
Psmd1	proteasome (prosome, macropain) 26S subunit, non-ATPase, 1
Pspc1	paraspeckle component 1
Ptgds	prostaglandin D2 synthase (brain)
Ptgr2	prostaglandin reductase 2
Ptms	parathymosin
Ptprk	protein tyrosine phosphatase, receptor type, K, extracellular region
Puf60	poly-U binding splicing factor 60
Pvrl1	poliovirus receptor-related 1
R3hdm2	R3H domain containing 2
Rab1b	RAB1B, member RAS oncogene family

Rab3gap2	RAB3 GTPase activating protein subunit 2
Raly	RNA binding protein, autoantigenic (hnRNP-associated with lethal yellow homolog (mouse))
Ramp2	receptor (G protein-coupled) activity modifying protein 2
Ranbp2	RAN binding protein 2
Ranbp6	RAN binding protein 6
Rasip1	Ras interacting protein 1
Rbbp5	retinoblastoma binding protein 5
Rbm15b	RNA binding motif protein 15B
Reps1	RALBP1 associated Eps domain containing 1
Rev3l	REV3-like, catalytic subunit of DNA polymerase zeta (yeast)
RGD1304884	similar to RIKEN cDNA 6430548M08
RGD1305793	similar to hypothetical protein FLJ20154
RGD1306151	similar to hypothetical protein DKFZp761D0211
RGD1306556	similar to hypothetical protein A530094D01
RGD1307752	similar to RIKEN cDNA 1110008F13
RGD1308143	similar to D330021B20 protein
RGD1309079	similar to Ab2-095
RGD1309735	similar to CG14977-PA
RGD1560871	similar to plexin 1
RGD1561963	similar to Dedicator of cytokinesis protein 10 (Protein zizimin 3)
RGD1564964	similar to WD repeat domain 11 protein
RGD1565257	similar to zinc finger protein 650
RGD1565675	similar to RIKEN cDNA 2410022L05
Rgp1	RGP1 retrograde golgi transport homolog (S. cerevisiae)
Rilpl1	Rab interacting lysosomal protein-like 1
Rnd3	Rho family GTPase 3
Rnf126	ring finger protein 126
Rnf166	ring finger protein 166
Rpa1	replication protein A1
Rpia	ribose 5-phosphate isomerase A
Rpl36al	ribosomal protein L36a-like
Rps17	ribosomal protein S17
Rps21	ribosomal protein S21
Rps24	ribosomal protein S24
Rpsa	ribosomal protein SA
Rsbn1	round spermatid basic protein 1
Rsbn1l	round spermatid basic protein 1-like
RT1-CE16	RT1 class I, locus CE16
Rtkn	rhotekin
Rtp4	receptor (chemosensory) transporter protein 4
Rufy3	RUN and FYVE domain containing 3
Scamp5	secretory carrier membrane protein 5
Scgb1c1	secretoglobin, family 1C, member 1
Sdccag1	serologically defined colon cancer antigen 1
Sdccag3	serologically defined colon cancer antigen 3

Sec23a	Sec23 homolog A (S. cerevisiae)
Sec3l1	SEC3-like 1 (S. cerevisiae)
Sec61a1	Sec61 alpha 1 subunit (S. cerevisiae)
Sec63	SEC63 homolog (S. cerevisiae)
Sesn1	sestrin 1
Sfxn1	sideroflexin 1
Sgta	small glutamine-rich tetratricopeptide repeat (TPR)-containing, alpha
Sh3gl2	SH3-domain GRB2-like 2
Shank2	SH3 and multiple ankyrin repeat domains 2
Shc2	SHC (Src homology 2 domain containing) transforming protein 2
Slbp	stem-loop binding protein
Slc11a2	solute carrier family 11 (proton-coupled divalent metal ion transporters), member 2
Slc13a4	solute carrier family 13 (sodium/sulfate symporters), member 4
Slc27a4	solute carrier family 27 (fatty acid transporter), member 4
Slc39a7	solute carrier family 39 (zinc transporter), member 7
Slc9a1	solute carrier family 9 (sodium/hydrogen exchanger), member 1
Slco1a5	solute carrier organic anion transporter family, member 1a5
Smarca4	SWI/SNF related, matrix associated, actin dependent regulator of chromatin, subfamily a, member 4
Smarca5	SWI/SNF related, matrix associated, actin dependent regulator of chromatin, subfamily a, member 5
Smyd3	SET and MYND domain containing 3
Snap29	synaptosomal-associated protein 29
Snapc2	small nuclear RNA activating complex, polypeptide 2
Sncb	synuclein, beta
Snrpf	small nuclear ribonucleoprotein polypeptide F
Snrpg	small nuclear ribonucleoprotein polypeptide G
Snx16	sorting nexin 16
Snx27	sorting nexin family member 27
Sostdc1	sclerostin domain containing 1
Spns1	spinster homolog 1 (Drosophila)
Sptbn4	spectrin, beta, non-erythrocytic 4
Srek1ip1	SREK1-interacting protein 1
Srf	serum response factor (c-fos serum response element-binding transcription factor)
Srrm1	serine/arginine repetitive matrix 1
Srrm2	serine/arginine repetitive matrix 2
Srsf11	serine/arginine-rich splicing factor 11
Ssr1	signal sequence receptor, alpha
Stk11	serine/threonine kinase 11
Stk25	serine/threonine kinase 25
Stk39	serine threonine kinase 39
Stx18	syntaxin 18
Stx6	syntaxin 6
Sv2b	synaptic vesicle glycoprotein 2b
Sybu	syntabulin (syntaxin-interacting)
Synj2bp	synaptojanin 2 binding protein
Syt16	synaptotagmin XVI

Syt7	synaptotagmin VII
Taf1d	TATA box binding protein (Tbp)-associated factor, RNA polymerase I, D
Taf9b	TAF9B RNA polymerase II, TATA box binding protein (TBP)-associated factor
Tars2	threonyl-tRNA synthetase 2, mitochondrial (putative)
Tbc1d15	TBC1 domain family, member 15
Tbl1x	transducin (beta)-like 1 X-linked
Tcf12	transcription factor 12
Tcn2	transcobalamin 2
Tcta	T-cell leukemia translocation altered gene
Tef	thyrotrophic embryonic factor
Thoc2	THO complex 2
Timm13	translocase of inner mitochondrial membrane 13 homolog (yeast)
Tlx3	T-cell leukemia, homeobox 3
Tm9sf4	transmembrane 9 superfamily protein member 4
Tmem33	transmembrane protein 33
Tmx4	thioredoxin-related transmembrane protein 4
Tollip	toll interacting protein
Tpm3	tropomyosin 3, gamma
Tpr	translocated promoter region (to activated MET oncogene)
Trim47	tripartite motif-containing 47
Trio	triple functional domain (PTPRF interacting)
Trpm7	transient receptor potential cation channel, subfamily M, member 7
Ttl	tubulin tyrosine ligase
Ttr	transthyretin
Txn1	thioredoxin 1
Ttyh1	tweety homolog 1 (Drosophila)
Uba5	ubiquitin-like modifier activating enzyme 5
Uba6	ubiquitin-like modifier activating enzyme 6
Ubl3	ubiquitin-like 3
Ubr2	ubiquitin protein ligase E3 component n-recognin 2
Ucma	upper zone of growth plate and cartilage matrix associated
Ugp2	UDP-glucose pyrophosphorylase 2
Upf3a	UPF3 regulator of nonsense transcripts homolog A (yeast)
Usp25	ubiquitin specific peptidase 25
Usp46	ubiquitin specific peptidase 46
Vps45	vacuolar protein sorting 45 homolog (S. cerevisiae)
Vps4a	vacuolar protein sorting 4 homolog A (S. cerevisiae)
Vrk1	vaccinia related kinase 1
Wbp1	WW domain binding protein 1
Wdr7	WD repeat domain 7
Whsc1l1	Wolf-Hirschhorn syndrome candidate 1-like 1 (human)
Wwc1	WW and C2 domain containing 1
Xylt2	xylosyltransferase II
Yaf2	YY1 associated factor 2
Yipf1	Yip1 domain family, member 1

Ythdf2	YTH domain family, member 2
Zfand5	zinc finger, AN1-type domain 5
Zfp26	zinc finger protein 26
Zfp329	zinc finger protein 329
Zfp523	zinc finger protein 523
Zfp61	zinc finger protein 61
Zfp709l2	zinc finger protein 709-like 2
Zmym6	zinc finger, MYM-type 6
Zscan10	zinc finger and SCAN domain containing 10

Supplemetray Table 2. List of up- and downregulated genes in the hippocampal formation after chronic DPN treatment. P Adj, P Adjusted; FC, fold change.

UPREGULATED GENES

logFC	FC	*P* Adj	Symbol	Description
4.863	29.10	0.005	*Ttr*	transthyretin
3.991	15.89	0.013	*Cldn2*	claudin 2
3.143	8.834	0.031	*Kl*	Klotho
3.105	8.602	0.015	*Igfbp2*	insulin-like growth factor binding protein 2
2.852	7.219	0.017	*Scgb1c1*	secretoglobin, family 1C, member 1
2.730	6.635	0.014	*Folr1*	folate receptor 1 (adult)
2.670	6.366	0.018	*Prlr*	prolactin receptor
2.640	6.235	0.031	*Sostdc1*	sclerostin domain containing 1
2.325	5.009	0.008	*Snrpg*	small nuclear ribonucleoprotein polypeptide G
2.313	4.968	0.007	*Ptgds*	prostaglandin D2 synthase (brain)
2.305	4.940	0.032	*Igf2*	insulin-like growth factor 2
2.279	4.854	0.013	*Mlec*	malectin
2.185	4.547	0.006	*Sec63*	SEC63 homolog (S. cerevisiae)
2.173	4.509	0.047	*Mfrp*	membrane frizzled-related protein
2.136	4.397	0.006	*Sptbn4*	spectrin, beta, non-erythrocytic 4
2.068	4.193	0.013	*Mdk*	midkine
2.061	4.173	0.036	*Ace*	angiotensin I converting enzyme (peptidyl-dipeptidase A) 1
2.055	4.156	0.007	*Pvrl1*	poliovirus receptor-related 1
1.913	3.767	0.014	*Hnrnpf*	heterogeneous nuclear ribonucleoprotein F
1.911	3.760	0.040	*Slc13a4*	solute carrier family 13 (sodium/sulfate symporters), member 4
1.891	3.710	0.005	*Hist2h2aa3*	histone cluster 2, H2aa3
1.838	3.575	0.061	*F5*	coagulation factor V (proaccelerin, labile factor)
1.836	3.570	0.005	*Gigyf2*	GRB10 interacting GYF protein 2
1.833	3.562	0.005	*Ccar1*	cell division cycle and apoptosis regulator 1
1.827	3.548	0.011	*Sec3l1*	SEC3-like 1 (S. cerevisiae)
1.746	3.354	0.008	*Yipf1*	Yip1 domain family, member 1
1.745	3.351	0.005	*Mllt6*	myeloid/lymphoid or mixed-lineage leukemia (trithorax homolog); translocated to, 6
1.721	3.296	0.007	*Arhgap32*	Rho GTPase activating protein 32
1.680	3.204	0.030	*Rpsa*	ribosomal protein SA
1.669	3.180	0.006	*Tbl1x*	transducin (beta)-like 1 X-linked
1.658	3.155	0.010	*Stx6*	syntaxin 6
1.655	3.150	0.078	*Kcne2*	potassium voltage-gated channel, Isk-related family, member 2
1.655	3.149	0.012	*Pds5b*	PDS5, regulator of cohesion maintenance, homolog B (S. cerevisiae)
1.627	3.089	0.006	*Klc3*	kinesin light chain 3
1.611	3.055	0.006	*Ssr1*	signal sequence receptor, alpha
1.590	3.010	0.003	*Inpp5f*	inositol polyphosphate-5-phosphatase F
1.586	3.003	0.009	*Phip*	Pleckstrin homology domain interacting protein
1.577	2.984	0.008	*Agtpbp1*	ATP/GTP binding protein 1
1.571	2.972	0.016	*Hapln2*	hyaluronan and proteoglycan link protein 2
1.571	2.971	0.020	*Fibcd1*	fibrinogen C domain containing 1
1.570	2.970	0.010	*Mrps27*	mitochondrial ribosomal protein S27
1.561	2.950	0.010	*Nupl1*	nucleoporin like 1
1.536	2.900	0.010	*Baz2b*	bromodomain adjacent to zinc finger domain, 2B
1.528	2.883	0.008	*Adam11*	ADAM metallopeptidase domain 11
1.527	2.881	0.014	*Hk1*	hexokinase 1
1.501	2.830	0.005	*Prkag2*	protein kinase, AMP-activated, gamma 2 non-catalytic subunit
1.499	2.826	0.009	*Slc9a1*	solute carrier family 9 (sodium/hydrogen exchanger), member 1
1.498	2.824	0.010	*Pcbd2*	pterin 4 alpha carbinolamine dehydratase/dimerization cofactor of hepatocyte nuclear factor 1
1.484	2.797	0.006	*Gtpbp6*	GTP binding protein 6 (putative)
1.483	2.796	0.005	*Lrrc4b*	leucine rich repeat containing 4B

2.794	0.006	*Acbd4*	acyl-CoA binding domain containing 4
2.792	0.010	*Atp5l*	ATP synthase, H+ transporting, mitochondrial Fo complex, subunit G
2.782	0.024	*Eif3d*	eukaryotic translation initiation factor 3, subunit D
2.778	0.049	*Col8a2*	collagen, type VIII, alpha 2
2.734	0.009	*Adcyap1r1*	adenylate cyclase activating polypeptide 1 receptor 1
2.731	0.007	*Stk25*	serine/threonine kinase 25
2.719	0.006	*Pbx1*	pre-B-cell leukemia homeobox 1
2.717	0.024	*Pgrmc2*	progesterone receptor membrane component 2
2.716	0.005	*Amt*	aminomethyltransferase
2.698	0.053	*Slco1a5*	solute carrier organic anion transporter family, member 1a5
2.695	0.011	*Pfn1*	profilin 1
2.681	0.033	*Slc39a7*	solute carrier family 39 (zinc transporter), member 7
2.676	0.010	*Tm9sf4*	transmembrane 9 superfamily protein member 4
2.676	0.005	*Myt1*	myelin transcription factor 1
2.672	0.030	*Prpf3*	PRP3 pre-mRNA processing factor 3 homolog (S. cerevisiae)
2.660	0.008	*Cdk10*	cyclin-dependent kinase 10
2.657	0.009	*Icam5*	intercellular adhesion molecule 5, telencephalin
2.648	0.001	*Sdccag3*	serologically defined colon cancer antigen 3
2.643	0.015	*Clptm1l*	CLPTM1-like
2.632	0.015	*Ubr2*	ubiquitin protein ligase E3 component n-recognin 2
2.631	0.007	*Sncb*	synuclein, beta
2.625	0.017	*Pabpn1*	poly(A) binding protein, nuclear 1
2.616	0.009	*Abcc1*	ATP-binding cassette, subfamily C (CFTR/MRP), member 1
2.615	0.038	*Raly*	RNA binding protein, autoantigenic (hnRNP-associated with lethal yellow homolog (mouse))
2.612	0.015	*RT1-CE16*	RT1 class I, locus CE16
2.607	0.007	*Fnbp4*	formin binding protein 4
2.597	0.021	*Arfgap1*	ADP-ribosylation factor GTPase activating protein 1
2.595	0.008	*Mbp*	myelin basic protein
2.593	0.005	*Taf1d*	TATA box binding protein (Tbp)-associated factor, RNA polymerase I, D
2.593	0.011	*LOC688786*	similar to CG14483-PA
2.588	0.011	*Srsf11*	serine/arginine-rich splicing factor 11
2.586	0.014	*Alkbh*	alkB, alkylation repair homolog (E. coli)
2.586	0.009	*Lrsam1*	leucine rich repeat and sterile alpha motif containing 1
2.584	0.014	*Rab1b*	RAB1B, member RAS oncogene family
2.578	0.011	*Gltscr1*	glioma tumor suppressor candidate region gene 1
2.553	0.013	*Xylt2*	xylosyltransferase II
2.553	0.070	*Ephb3*	Eph receptor B3
2.539	0.020	*Ncor1*	nuclear receptor co-repressor 1
2.535	0.011	*Mcu*	mitochondrial calcium uniporter
2.534	0.007	*Irak1*	interleukin-1 receptor-associated kinase 1
2.530	0.022	*Tpm3*	tropomyosin 3, gamma
2.521	0.007	*LOC301124*	hypothetical LOC301124
2.517	0.008	*Mapk8ip2*	mitogen-activated protein kinase 8 interacting protein 2
2.513	0.021	*Calml4*	calmodulin-like 4
2.508	0.007	*Mkl1*	megakaryoblastic leukemia (translocation) 1
2.507	0.015	*LOC100365679*	DENN/MADD domain containing 5B-like
2.502	0.011	*Rps21*	ribosomal protein S21
2.498	0.008	*Dlgap3*	discs, large (Drosophila) homolog-associated protein 3
2.497	0.008	*Sh3gl2*	SH3-domain GRB2-like 2
2.497	0.028	*Mau2*	Mau2 chromatid cohesion factor homolog (C. elegans)
2.493	0.022	*Pitpnm2*	phosphatidylinositol transfer protein, membrane-associated 2
2.492	0.011	*Immt*	inner membrane protein, mitochondrial
2.483	0.009	*Srf*	serum response factor (c-fos serum response element-binding transcription factor)

2.466	0.006	*Shank2*	SH3 and multiple ankyrin repeat domains 2
2.449	0.015	*Ash1l*	ash1 (absent, small, or homeotic)-like (Drosophila)
2.448	0.021	*Map7d2*	MAP7 domain containing 2
2.447	0.017	*Pnmal2*	PNMA-like 2
2.439	0.016	*Dos*	downstream of Stk11
2.438	0.015	*LOC294154*	similar to chromosome 6 open reading frame 106 isoform a
2.423	0.008	*Tcf12*	transcription factor 12
2.420	0.017	*Anapc11*	anaphase promoting complex subunit 11
2.418	0.008	*Ppp1r1a*	protein phosphatase 1, regulatory (inhibitor) subunit 1A
2.415	0.017	*Cacna1a*	calcium channel, voltage-dependent, P/Q type, alpha 1A subunit
2.413	0.023	*Nova2*	neuro-oncological ventral antigen 2
2.411	0.026	*Grm1*	glutamate receptor, metabotropic 1
2.406	0.022	*Ptms*	parathymosin
2.401	0.009	*Dtnbp1*	distrobrevin binding protein 1
2.398	0.006	*Grk6*	G protein-coupled receptor kinase 6
2.398	0.026	*Smarca4*	SWI/SNF related, matrix associated, actin dependent regulator of chromatin, subfamily a, 4
2.396	0.016	*Bloc1s1*	biogenesis of lysosomal organelles complex-1, subunit 1
2.395	0.006	*Ranbp6*	RAN binding protein 6
2.394	0.023	*Upf3a*	UPF3 regulator of nonsense transcripts homolog A (yeast)
2.391	0.011	*Rps17*	ribosomal protein S17
2.386	0.008	*Csnk2a2*	casein kinase 2, alpha prime polypeptide
2.384	0.017	*Pou3f3*	POU class 3 homeobox 3
2.375	0.008	*RGD1309735*	similar to CG14977-PA
2.374	0.005	*Akip1*	A kinase (PRKA) interacting protein 1
2.368	0.006	*Rps24*	ribosomal protein S24
2.367	0.006	*Cldn12*	claudin 12
2.365	0.014	*Fbxl6*	F-box and leucine-rich repeat protein 6
2.365	0.016	*RGD1304884*	similar to RIKEN cDNA 6430548M08
2.364	0.008	*Cdv3*	carnitine deficiency-associated gene expressed in ventricle 3 homolog (mouse)
2.358	0.008	*Mapk10*	mitogen activated protein kinase 10
2.344	0.006	*Rnf126*	ring finger protein 126
2.344	0.007	*Cd59*	CD59 molecule, complement regulatory protein
2.343	0.018	*Isca1*	iron-sulfur cluster assembly 1 homolog (S. cerevisiae)
2.343	0.022	*R3hdm2*	R3H domain containing 2
2.342	0.009	*Plekho1*	pleckstrin homology domain containing, family O member 1
2.339	0.021	*Epn2*	epsin 2
2.339	0.007	*Vps45*	vacuolar protein sorting 45 homolog (S. cerevisiae)
2.337	0.069	*Htr2c*	5-hydroxytryptamine (serotonin) receptor 2C
2.335	0.007	*Snap29*	synaptosomal-associated protein 29
2.335	0.010	*Fnbp1*	formin binding protein 1
2.330	0.014	*Hnrpd*	heterogeneous nuclear ribonucleoprotein D
2.328	0.008	*Sec61a1*	Sec61 alpha 1 subunit (S. cerevisiae)
2.323	0.020	*Pes1*	pescadillo homolog 1, containing BRCT domain (zebrafish)
2.317	0.018	*Mef2bnb*	MEF2B neighbor
2.316	0.018	*Cntn2*	contactin 2 (axonal)
2.315	0.016	*Hsp90ab1*	heat shock protein 90 alpha (cytosolic), class B member 1
2.311	0.023	*Prelid1*	PRELI domain containing 1
2.311	0.010	*Gga3*	golgi associated, gamma adaptin ear containing, ARF binding protein 3
2.300	0.012	*Timm13*	translocase of inner mitochondrial membrane 13 homolog (yeast)
2.299	0.010	*Pdlim5*	PDZ and LIM domain 5
2.295	0.018	*Nfyc*	nuclear transcription factor-Y gamma
2.294	0.077	*Fmod*	fibromodulin
2.292	0.011	*Reps1*	RALBP1 associated Eps domain containing 1

2.289	0.019	*Tmx4*	thioredoxin-related transmembrane protein 4
2.288	0.007	*Yaf2*	YY1 associated factor 2
2.288	0.012	*Ramp2*	receptor (G protein-coupled) activity modifying protein 2
2.286	0.009	*Pias1*	protein inhibitor of activated STAT, 1
2.283	0.023	*Ensa*	endosulfine alpha
2.281	0.007	*LOC100294508*	dyslexia susceptibility 2-like
2.279	0.020	*Mast3*	microtubule associated serine/threonine kinase 3
2.277	0.022	*Uba5*	ubiquitin-like modifier activating enzyme 5
2.274	0.005	*Klf13*	Kruppel-like factor 13
2.273	0.006	*Hip1*	huntingtin interacting protein 1
2.270	0.008	*Cerk*	ceramide kinase
2.270	0.006	*Dpp8*	dipeptidylpeptidase 8
2.270	0.015	*Fkbp1a*	FK506 binding protein 1a
2.269	0.008	*Ptprk*	protein tyrosine phosphatase, receptor type, K, extracellular region
2.269	0.013	*Psmd1*	proteasome (prosome, macropain) 26S subunit, non-ATPase, 1
2.268	0.012	*Kif1a*	kinesin family member 1A
2.268	0.009	*Stk39*	serine threonine kinase 39
2.267	0.011	*LOC100360606*	zinc finger, RAN-binding domain containing 1 protein-like
2.263	0.010	*Maged2*	melanoma antigen, family D, 2
2.261	0.007	*Smyd3*	SET and MYND domain containing 3
2.261	0.010	*Srrm1*	serine/arginine repetitive matrix 1
2.259	0.015	*Eif4g1*	eukaryotic translation initiation factor 4 gamma, 1
2.249	0.006	*Cnot6l*	CCR4-NOT transcription complex, subunit 6-like
2.247	0.031	*Nacad*	NAC alpha domain containing
2.240	0.043	*Ddit3*	DNA-damage inducible transcript 3
2.239	0.008	*Txn1*	thioredoxin 1
2.239	0.006	*Rnf166*	ring finger protein 166
2.237	0.007	*A2m*	alpha-2-macroglobulin
2.235	0.007	*Erbb4*	v-erb-a erythroblastic leukemia viral oncogene homolog 4 (avian)
2.234	0.015	*Tars2*	threonyl-tRNA synthetase 2, mitochondrial (putative)
2.234	0.008	*RGD1565675*	similar to RIKEN cDNA 2410022L05
2.233	0.068	*Aqp1*	aquaporin 1
2.233	0.010	*Pik3ca*	phosphoinositide-3-kinase, catalytic, alpha polypeptide
2.232	0.019	*Calm3*	calmodulin 3
2.232	0.008	*Nip30*	NEFA-interacting nuclear protein NIP30
2.232	0.006	*Mapre1*	microtubule-associated protein, RP/EB family, member 1
2.230	0.007	*Syt7*	synaptotagmin VII
2.228	0.017	*Atox1*	ATX1 antioxidant protein 1 homolog (yeast)
2.227	0.011	*Apc2*	adenomatosis polyposis coli 2
2.227	0.009	*Ctdp1*	CTD (carboxy-terminal domain, RNA polymerase II, polypeptide A) phosphatase, subunit 1
2.226	0.024	*LOC686944*	similar to Zinc finger protein 45 (BRC1744)
2.226	0.015	*Nbr1*	neighbor of Brca1 gene 1
2.219	0.020	*Ank3*	ankyrin 3, node of Ranvier
2.216	0.013	*Cldn11*	claudin 11
2.215	0.013	*Fkbp2*	FK506 binding protein 2
2.213	0.011	*Gnb5*	guanine nucleotide binding protein (G protein), beta 5
2.210	0.020	*Ddx27*	DEAD (Asp-Glu-Ala-Asp) box polypeptide 27
2.209	0.023	*Bcl2l1*	Bcl2-like 1
2.209	0.014	*Pdrg1*	p53 and DNA damage regulated 1
2.208	0.022	*Dnmt3a*	DNA (cytosine-5-)-methyltransferase 3 alpha
2.204	0.012	*Slc27a4*	solute carrier family 27 (fatty acid transporter), member 4
2.203	0.008	*Hcfc1r1*	host cell factor C1 regulator 1 (XPO1-dependent)
2.203	0.009	*Snapc2*	small nuclear RNA activating complex, polypeptide 2

2.200	0.101	*Otx2*	orthodenticle homeobox 2
2.200	0.022	*Ddx49*	DEAD (Asp-Glu-Ala-Asp) box polypeptide 49
2.195	0.006	*Sfxn1*	sideroflexin 1
2.195	0.006	*Abhd8*	abhydrolase domain containing 8
2.192	0.006	*Kctd1*	potassium channel tetramerisation domain containing 1
2.190	0.037	*Arl6ip4*	ADP-ribosylation-like factor 6 interacting protein 4
2.189	0.018	*Tpr*	translocated promoter region (to activated MET oncogene)
2.188	0.029	*Mars*	methionine-tRNA synthetase
2.183	0.011	*Ndufa12*	NADH dehydrogenase (ubiquinone) 1 alpha subcomplex, 12
2.182	0.019	*Elovl6*	ELOVL fatty acid elongase 6
2.181	0.006	*Zfp523*	zinc finger protein 523
2.174	0.016	*Nicn1*	nicolin 1
2.172	0.011	*Fxyd1*	FXYD domain-containing ion transport regulator 1
2.172	0.031	*Ttyh1*	tweety homolog 1 (Drosophila)
2.171	0.007	*Snx27*	sorting nexin family member 27
2.170	0.026	*Nucks1*	nuclear casein kinase and cyclin-dependent kinase substrate 1
2.169	0.011	*Akap2*	A kinase (PRKA) anchor protein 2
2.168	0.007	*LOC100188932*	dolichyl-diphosphooligosaccharide--protein glycosyltransferase subunit 4
2.168	0.028	*Rpl36al*	ribosomal protein L36a-like
2.167	0.006	*Tef*	thyrotrophic embryonic factor
2.164	0.013	*Aig1*	androgen-induced 1
2.159	0.037	*Gns*	glucosamine (N-acetyl)-6-sulfatase
2.156	0.006	*Atp2a2*	ATPase, Ca++ transporting, cardiac muscle, slow twitch 2
2.152	0.006	*Sv2b*	synaptic vesicle glycoprotein 2b
2.152	0.034	*Thoc2*	THO complex 2
2.148	0.022	*Padi2*	peptidyl arginine deiminase, type II
2.146	0.008	*Gtf3c6*	general transcription factor IIIC, polypeptide 6, alpha
2.146	0.017	*Paf1*	Paf1, RNA polymerase II associated factor, homolog (S. cerevisiae)
2.146	0.005	*Chrm4*	cholinergic receptor, muscarinic 4
2.146	0.009	*Kcnc2*	potassium voltage gated channel, Shaw-related subfamily, member 2
2.146	0.015	*Itsn2*	intersectin 2
2.144	0.013	*Copg*	coatomer protein complex, subunit gamma
2.143	0.012	*Nat14*	N-acetyltransferase 14
2.142	0.025	*Spns1*	spinster homolog 1 (Drosophila)
2.142	0.014	*RGD1306556*	similar to hypothetical protein A530094D01
2.142	0.010	*Stx18*	syntaxin 18
2.140	0.013	*Slc11a2*	solute carrier family 11 (proton-coupled divalent metal ion transporters), member 2
2.137	0.012	*Sgta*	small glutamine-rich tetratricopeptide repeat (TPR)-containing, alpha
2.137	0.011	*Pls3*	plastin 3
2.134	0.014	*Pnisr*	PNN-interacting serine/arginine-rich protein
2.134	0.019	*Kcnd3*	potassium voltage-gated channel, Shal-related subfamily, member 3
2.134	0.010	*Pde4d*	phosphodiesterase 4D, cAMP-specific
2.133	0.018	*Dcaf6*	DDB1 and CUL4 associated factor 6
2.131	0.017	*Hnrnpu*	heterogeneous nuclear ribonucleoprotein U
2.128	0.017	*Pbxip1*	pre-B-cell leukemia homeobox interacting protein 1
2.127	0.015	*Mrpl42*	mitochondrial ribosomal protein L42
2.127	0.016	*Lace1*	lactation elevated 1
2.127	0.016	*Kcna6*	potassium voltage gated channel, shaker related subfamily, member 6
2.124	0.018	*Kcnma1*	potassium large conductance calcium-activated channel, subfamily M, alpha member 1
2.124	0.027	*Ddx3x*	DEAD (Asp-Glu-Ala-Asp) box polypeptide 3, X-linked
2.121	0.007	*Nedd4*	neural precursor cell expressed, developmentally down-regulated 4
2.119	0.011	*Dohh*	deoxyhypusine hydroxylase/monooxygenase
2.112	0.005	*Nfia*	nuclear factor I/A

2.111	0.005	*Eml4*	echinoderm microtubule associated protein like 4
2.111	0.011	*Synj2bp*	synaptojanin 2 binding protein
2.108	0.026	*Arl10*	ADP-ribosylation factor-like 10
2.107	0.013	*Mxd4*	Max dimerization protein 4
2.107	0.022	*LOC681825*	similar to Prefoldin subunit 3 (Von Hippel-Lindau-binding protein 1, VBP-1)
2.106	0.022	*Klc1*	kinesin light chain 1
2.105	0.017	*LOC683519*	similar to ribosomal protein, mitochondrial, S22
2.105	0.007	*Srek1ip1*	SREK1-interacting protein 1
2.105	0.015	*Pnkd*	paroxysmal nonkinesiogenic dyskinesia
2.104	0.010	*Nedd9*	neural precursor cell expressed, developmentally down-regulated 9
2.101	0.007	*Eif3s6ip*	eukaryotic translation initiation factor 3, subunit 6 interacting protein
2.100	0.010	*Eif3e*	eukaryotic translation initiation factor 3, subunit E
2.100	0.015	*Chst2*	carbohydrate sulfotransferase 2
2.098	0.013	*Ctsh*	cathepsin H
2.098	0.007	*Ptgr2*	prostaglandin reductase 2
2.097	0.010	*Snrpf*	small nuclear ribonucleoprotein polypeptide F
2.096	0.012	*Vps4a*	vacuolar protein sorting 4 homolog A (S. cerevisiae)
2.095	0.011	*Dag1*	dystroglycan 1 (dystrophin-associated glycoprotein 1)
2.093	0.015	*LOC688018*	similar to SH3-domain binding protein 3
2.093	0.018	*LOC501091*	similar to Discs large homolog 5 (Placenta and prostate DLG) (Discs large protein P-dlg)
2.091	0.019	*Wbp1*	WW domain binding protein 1
2.090	0.011	*Sybu*	syntabulin (syntaxin-interacting)
2.090	0.006	*Commd6*	COMM domain containing 6
2.090	0.005	*Cxcr4*	chemokine (C-X-C motif) receptor 4
2.088	0.049	*Nf2*	neurofibromin 2 (merlin)
2.088	0.010	*Amigo2*	adhesion molecule with Ig like domain 2
2.088	0.013	*Fam18a*	family with sequence similarity 18, member A
2.087	0.009	*RGD1561963*	similar to Dedicator of cytokinesis protein 10 (Protein zizimin 3)
2.085	0.020	*Pla2g6*	phospholipase A2, group VI (cytosolic, calcium-independent)
2.082	0.041	*RGD1560871*	similar to plexin 1
2.081	0.025	*Map7*	microtubule-associated protein 7
2.080	0.017	*Cbfa2t3*	core-binding factor, runt domain, alpha subunit 2; translocated to, 3
2.079	0.014	*Copz1*	coatomer protein complex, subunit zeta 1
2.079	0.009	*Gng12*	guanine nucleotide binding protein (G protein), gamma 12
2.077	0.023	*LOC691468*	similar to Zinc finger protein 84 (Zinc finger protein HPF2)
2.074	0.006	*Srrm2*	serine/arginine repetitive matrix 2
2.073	0.018	*Pltp*	phospholipid transfer protein
2.070	0.011	*Ttl*	tubulin tyrosine ligase
2.070	0.024	*Puf60*	poly-U binding splicing factor 60
2.069	0.011	*Prrc2c*	proline-rich coiled-coil 2C
2.069	0.017	*Khsrp*	KH-type splicing regulatory protein
2.067	0.008	*Cpsf3*	cleavage and polyadenylation specific factor 3, 73kDa
2.066	0.008	*Cdk7*	cyclin-dependent kinase 7
2.065	0.015	*Lsm7*	LSM7 homolog, U6 small nuclear RNA associated (S. cerevisiae)
2.063	0.006	*Rab3gap2*	RAB3 GTPase activating protein subunit 2
2.063	0.008	*Rbbp5*	retinoblastoma binding protein 5
2.060	0.015	*Atp5e*	ATP synthase, H+ transporting, mitochondrial F1 complex, epsilon subunit
2.059	0.013	*Acadvl*	acyl-CoA dehydrogenase, very long chain
2.059	0.014	*Sdccag1*	serologically defined colon cancer antigen 1
2.057	0.008	*Rtkn*	rhotekin
2.056	0.005	*Hars2l*	histidyl-tRNA synthetase 2-like
2.054	0.011	*Pdia4*	protein disulfide isomerase family A, member 4
2.054	0.008	*Tcta*	T-cell leukemia translocation altered gene

2.054	0.007	*Ide*	insulin degrading enzyme
2.052	0.008	*Polr2b*	polymerase (RNA) II (DNA directed) polypeptide B
2.049	0.008	*Wdr7*	WD repeat domain 7
2.047	0.028	*Mrps16*	mitochondrial ribosomal protein S16
2.047	0.062	*Enpp2*	ectonucleotide pyrophosphatase/phosphodiesterase 2
2.045	0.009	*Gapdh*	glyceraldehyde-3-phosphate dehydrogenase
2.045	0.009	*Pdzd4*	PDZ domain containing 4
2.044	0.014	*Ncln*	nicalin
2.043	0.010	*Fam183b*	family with sequence similarity 183, member B
2.043	0.012	*Zfp61*	zinc finger protein 61
2.042	0.005	*Actr2*	ARP2 actin-related protein 2 homolog (yeast)
2.041	0.014	*Ndufv3*	NADH dehydrogenase (ubiquinone) flavoprotein 3
2.041	0.021	*Med1*	mediator complex subunit 1
2.039	0.018	*Gpatch4*	G patch domain containing 4
2.037	0.022	*Lgals1*	lectin, galactoside-binding, soluble, 1
2.037	0.019	*Fermt2*	fermitin family member 2
2.036	0.010	*Kcnj3*	potassium inwardly-rectifying channel, subfamily J, member 3
2.036	0.010	*RGD1565257*	similar to zinc finger protein 650
2.034	0.009	*Nolc1*	nucleolar and coiled-body phosphoprotein 1
2.034	0.013	*B9d1*	B9 protein domain 1
2.032	0.008	*Sesn1*	sestrin 1
2.030	0.005	*Akt1s1*	AKT1 substrate 1 (proline-rich)
2.027	0.009	*Agpat3*	1-acylglycerol-3-phosphate O-acyltransferase 3
2.025	0.028	*Pank2*	pantothenate kinase 2 (Hallervorden-Spatz syndrome)
2.025	0.019	*Edc4*	enhancer of mRNA decapping 4
2.024	0.008	*Ubl3*	ubiquitin-like 3
2.023	0.011	*Dnaja3*	DnaJ (Hsp40) homolog, subfamily A, member 3
2.023	0.009	*RGD1307752*	similar to RIKEN cDNA 1110008F13
2.022	0.017	*Rgp1*	RGP1 retrograde golgi transport homolog (S. cerevisiae)
2.021	0.058	*Zfp709l2*	zinc finger protein 709-like 2
2.018	0.013	*Il16*	interleukin 16
2.016	0.033	*Ctdnep1*	CTD nuclear envelope phosphatase 1
2.015	0.009	*Ddx6*	DEAD (Asp-Glu-Ala-Asp) box polypeptide 6
2.014	0.008	*Dda1*	DET1 and DDB1 associated 1
2.014	0.005	*Mgea5*	meningioma expressed antigen 5 (hyaluronidase)
2.013	0.014	*Gas5*	growth arrest specific 5
2.013	0.022	*Nudcd3*	NudC domain containing 3
2.013	0.016	*Chd2*	chromodomain helicase DNA binding protein 2
2.012	0.006	*Dlgap1*	discs, large (Drosophila) homolog-associated protein 1
2.012	0.012	*LOC687029*	similar to differentially expressed in B16F10 1
2.011	0.013	*Scamp5*	secretory carrier membrane protein 5
2.011	0.011	*Mif*	macrophage migration inhibitory factor
2.010	0.023	*Hectd1*	HECT domain containing 1
2.010	0.012	*Rufy3*	RUN and FYVE domain containing 3
2.009	0.028	*RGD1306151*	similar to hypothetical protein DKFZp761D0211
2.008	0.005	*Mob1b*	MOB kinase activator 1B
2.007	0.009	*Chrm1*	cholinergic receptor, muscarinic 1
2.006	0.011	*Fars2*	phenylalanyl-tRNA synthetase 2, mitochondrial
2.006	0.013	*Mgll*	monoglyceride lipase
2.004	0.009	*Ep400*	E1A binding protein p400
2.004	0.008	*Tcn2*	transcobalamin 2
2.003	0.019	*Pfkfb2*	6-phosphofructo-2-kinase/fructose-2,6-biphosphatase 2
2.002	0.006	*Tollip*	toll interacting protein

1.001	2.001	0.013	*Aimp1*	aminoacyl tRNA synthetase complex-interacting multifunctional protein 1
1.000	2.000	0.012	*RGD1305793*	similar to hypothetical protein FLJ20154
1.000	2.000	0.011	*LOC690769*	similar to zinc ring finger protein 1

DOWNREGULATED GENES

logFC	FC	*P* Adj	Symbol	Description
-0.999	0.500	0.008	*Bdkrb2*	bradykinin receptor B2
-1.002	0.499	0.007	*Kctd4*	potassium channel tetramerisation domain containing 4
-1.002	0.499	0.013	*Gls*	glutaminase
-1.004	0.499	0.047	*Fndc3a*	fibronectin type III domain containing 3a
-1.006	0.498	0.014	*LOC100365089*	rCG50929-like
-1.007	0.498	0.045	*Rtp4*	receptor (chemosensory) transporter protein 4
-1.008	0.497	0.013	*Fam134b*	family with sequence similarity 134, member B
-1.011	0.496	0.007	*LOC100363114*	eukaryotic translation initiation factor 4 gamma, 3-like
-1.012	0.496	0.018	*Eif5a2*	eukaryotic translation initiation factor 5A2
-1.014	0.495	0.016	*Snx16*	sorting nexin 16
-1.014	0.495	0.007	*Dclk2*	doublecortin-like kinase 2
-1.015	0.495	0.008	*Trim47*	tripartite motif-containing 47
-1.016	0.494	0.011	*Elavl3*	ELAV (embryonic lethal, abnormal vision, Drosophila)-like 3 (Hu antigen C)
-1.016	0.494	0.024	*Uba6*	ubiquitin-like modifier activating enzyme 6
-1.016	0.494	0.008	*Chst11*	carbohydrate (chondroitin 4) sulfotransferase 11
-1.018	0.494	0.122	*Nek1*	NIMA (never in mitosis gene a)-related kinase 1
-1.018	0.494	0.013	*Csrnp2*	cysteine-serine-rich nuclear protein 2
-1.019	0.494	0.021	*Gca*	grancalcin
-1.019	0.493	0.007	*Gigyf1*	GRB10 interacting GYF protein 1
-1.020	0.493	0.010	*Rev3l*	REV3-like, catalytic subunit of DNA polymerase zeta (yeast)
-1.022	0.492	0.005	*Kcnk2*	potassium channel, subfamily K, member 2
-1.024	0.492	0.005	*Cmtm4*	CKLF-like MARVEL transmembrane domain containing 4
-1.025	0.492	0.006	*LOC680835*	similar to cullin 7
-1.031	0.490	0.007	*Asap1*	ArfGAP with SH3 domain, ankyrin repeat and PH domain 1
-1.031	0.490	0.032	*Ddx58*	DEAD (Asp-Glu-Ala-Asp) box polypeptide 58
-1.032	0.489	0.006	*Pspc1*	paraspeckle component 1
-1.033	0.489	0.007	*Mga*	MAX gene associated
-1.035	0.488	0.027	*Hif1a*	hypoxia-inducible factor 1, alpha subunit (basic helix-loop-helix transcription factor)
-1.035	0.488	0.007	*Arpp19*	cAMP-regulated phosphoprotein 19
-1.036	0.488	0.011	*Fam195a*	family with sequence similarity 195, member A
-1.036	0.488	0.074	*Smarca5*	SWI/SNF related, matrix associated, actin dependent regulator of chromatin, subfamily a, 5
-1.037	0.487	0.018	*Bckdha*	branched chain ketoacid dehydrogenase E1, alpha polypeptide
-1.039	0.487	0.007	*LOC682967*	similar to Protein disulfide-isomerase precursor (Thioredoxin domain-containing protein 10)
-1.046	0.484	0.016	*Neurod1*	neurogenic differentiation 1
-1.047	0.484	0.036	*RGD1308143*	similar to D330021B20 protein
-1.051	0.483	0.020	*RGD1309079*	similar to Ab2-095
-1.051	0.483	0.051	*Fos*	FBJ osteosarcoma oncogene
-1.052	0.482	0.005	*Usp46*	ubiquitin specific peptidase 46
-1.054	0.482	0.006	*LOC688549*	hypothetical protein LOC688549
-1.056	0.481	0.007	*Rilpl1*	Rab interacting lysosomal protein-like 1
-1.057	0.480	0.018	*Arhgef26*	Rho guanine nucleotide exchange factor (GEF) 26
-1.061	0.479	0.006	*Kbtbd2*	kelch repeat and BTB (POZ) domain containing 2
-1.069	0.477	0.015	*Ndfip2*	Nedd4 family interacting protein 2
-1.073	0.475	0.080	*Papolg*	poly(A) polymerase gamma
-1.075	0.475	0.015	*Commd10*	COMM domain containing 10
-1.081	0.473	0.019	*Rasip1*	Ras interacting protein 1
-1.083	0.472	0.041	*Fbrsl1*	fibrosin-like 1
-1.084	0.472	0.024	*Prcp*	prolylcarboxypeptidase (angiotensinase C)
-1.087	0.471	0.008	*Shc2*	SHC (Src homology 2 domain containing) transforming protein 2
-1.089	0.470	0.007	*Cox11*	COX11 cytochrome c oxidase assembly homolog (yeast)
-1.089	0.470	0.021	*Mob1a*	MOB kinase activator 1A
-1.092	0.469	0.080	*Frmd4b*	FERM domain containing 4B

0.468	0.021	*Whsc1l1*	Wolf-Hirschhorn syndrome candidate 1-like 1 (human)
0.467	0.013	*Zfp329*	zinc finger protein 329
0.467	0.010	*Plekhh1*	pleckstrin homology domain containing, family H (with MyTH4 domain) member 1
0.467	0.014	*Nasp*	nuclear autoantigenic sperm protein (histone-binding)
0.467	0.023	*Crebbp*	CREB binding protein
0.466	0.005	*Cdk13*	cyclin-dependent kinase 13
0.466	0.006	*Zfand5*	zinc finger, AN1-type domain 5
0.465	0.016	*Wwc1*	WW and C2 domain containing 1
0.463	0.029	*Ddx26b*	DEAD/H (Asp-Glu-Ala-Asp/His) box polypeptide 26B
0.462	0.006	*Lrp11*	low density lipoprotein receptor-related protein 11
0.461	0.010	*Nol4*	nucleolar protein 4
0.461	0.021	*LOC100361182*	armadillo repeat containing, X-linked 5
0.460	0.015	*Ncoa6*	nuclear receptor coactivator 6
0.459	0.011	*Ugp2*	UDP-glucose pyrophosphorylase 2
0.459	0.085	*Arl13b*	ADP-ribosylation factor-like 13B
0.458	0.011	*Grxcr1*	glutaredoxin, cysteine rich 1
0.457	0.011	*Ucma*	upper zone of growth plate and cartilage matrix associated
0.457	0.008	*Ccdc25*	coiled-coil domain containing 25
0.456	0.009	*Dld*	dihydrolipoamide dehydrogenase
0.456	0.007	*Stk11*	serine/threonine kinase 11
0.456	0.011	*Kat6a*	K(lysine) acetyltransferase 6A
0.455	0.015	*Ythdf2*	YTH domain family, member 2
0.454	0.010	*Ces5a*	carboxylesterase 5A
0.454	0.027	*Fubp3*	far upstream element (FUSE) binding protein 3
0.454	0.005	*Zscan10*	zinc finger and SCAN domain containing 10
0.451	0.008	*Elfn2-ps1*	extracellular leucine-rich repeat and fibronectin type III domain containing 2, pseudogene
0.451	0.009	*LOC681251*	hypothetical protein LOC681251
0.450	0.006	*C2cd2l*	C2 calcium-dependent domain containing 2-like
0.446	0.013	*Rpa1*	replication protein A1
0.445	0.006	*Ldb2*	LIM domain binding 2
0.442	0.006	*Zmym6*	zinc finger, MYM-type 6
0.442	0.006	*Pabpc1*	poly(A) binding protein, cytoplasmic 1
0.442	0.012	*Amn1*	antagonist of mitotic exit network 1 homolog (S. cerevisiae)
0.440	0.009	*Camsap2*	calmodulin regulated spectrin-associated protein family, member 2
0.440	0.014	*Cdkn2c*	cyclin-dependent kinase inhibitor 2C (p18, inhibits CDK4)
0.439	0.031	*Abca1*	ATP-binding cassette, subfamily A (ABC1), member 1
0.437	0.013	*LOC684258*	similar to coiled-coil-helix-coiled-coil-helix domain containing 7
0.432	0.014	*Syt16*	synaptotagmin XVI
0.429	0.011	*Abcd2*	ATP-binding cassette, subfamily D (ALD), member 2
0.426	0.005	*Rsbn1*	round spermatid basic protein 1
0.424	0.020	*Kdm6a*	lysine (K)-specific demethylase 6A
0.422	0.008	*Ate1*	arginyltransferase 1
0.422	0.049	*Taf9b*	TAF9B RNA polymerase II, TATA box binding protein (TBP)-associated factor
0.421	0.008	*Mex3b*	mex3 homolog B (C. elegans)
0.419	0.006	*Dck*	deoxycytidine kinase
0.418	0.006	*LOC689298*	hypothetical protein LOC689298
0.418	0.045	*Psip1*	PC4 and SFRS1 interacting protein 1
0.417	0.010	*Tlx3*	T-cell leukemia, homeobox 3
0.416	0.042	*Tmem33*	transmembrane protein 33
0.416	0.007	*Cnfn*	cornifelin
0.412	0.007	*Gkap1*	G kinase anchoring protein 1
0.407	0.005	*Rbm15b*	RNA binding motif protein 15B
0.406	0.012	*Rnd3*	Rho family GTPase 3

0.405	0.041	*Dhx36*	DEAH (Asp-Glu-Ala-His) box polypeptide 36
0.401	0.013	*Ints6*	integrator complex subunit 6
0.401	0.060	*Zfp26*	zinc finger protein 26
0.399	0.013	*Rsbn1l*	round spermatid basic protein 1-like
0.398	0.028	*Lin7c*	lin-7 homolog C (C. elegans)
0.392	0.008	*Kpna3*	karyopherin alpha 3
0.391	0.026	*Usp25*	ubiquitin specific peptidase 25
0.390	0.008	*Vrk1*	vaccinia related kinase 1
0.389	0.006	*Rpia*	ribose 5-phosphate isomerase A
0.384	0.013	*Trio*	triple functional domain (PTPRF interacting)
0.383	0.015	*Pik3ca*	phosphoinositide-3-kinase, catalytic, alpha polypeptide
0.376	0.011	*Pacrgl*	PARK2 co-regulated-like
0.373	0.010	*Tbc1d15*	TBC1 domain family, member 15
0.366	0.025	*Trpm7*	transient receptor potential cation channel, subfamily M, member 7
0.366	0.011	*Grm8*	glutamate receptor, metabotropic 8
0.363	0.011	*Sec23a*	Sec23 homolog A (S. cerevisiae)
0.352	0.007	*Ranbp2*	RAN binding protein 2
0.337	0.009	*Klf3*	Kruppel-like factor 3 (basic)
0.328	0.018	*Fam91a1*	family with sequence similarity 91, member A1
0.312	0.007	*Acyp2*	acylphosphatase 2, muscle type
0.265	0.013	*Mrpl51*	mitochondrial ribosomal protein L51
0.220	0.020	*Slbp*	stem-loop binding protein

Supplementary Table 3 Functional annotation clustering. DPN-regulated genes were analyzed by DAVID v6.7 (12) bioinformatics tool. Functional annotation clustering was performed at high stringency. The annotation clusters were ranked based on their enrichment scores (ES). Top clusters indicate modulation of transcription, mRNA splicing, protein transport and neurotransmission.

Cluster	ES	Count	*P*	Benjamini
**Membrane-enclosed lumen**<br>Atp5e, Bcl2l, Crebbp, Ctdp1, Dnmt3a, Dnaja3, ep400, Fos, Mga, Paf1, Taf9b, Acadvl, A2m, Anapc11, Bckd4a, Calm1, F5, Cpox, Cdk7, Dld, Eif3sbip, Eif3e, Gtf3c6, Gls, Hnrnpf, Hif1a, Ide, Ints6, Med1, Mrpl51, Mrps16, Nf2, Ncor1, Ncoa6, Nfyc, Nolc1, Pspc1, Fars2, Polr2b, Pbx1, Pdia4, Rpa1, Rbbp5, Pes1, Snrpf, Sptbn4, Tars2, Tcf12, Tbl1x, Timm13, Tpr, Utx	4.39	53	2.1E-05	8.2E-03
**mRNA metabolic process**<br>Bcl2l, Khsrp, Sfrs12ip1, Ccar1, Cpsf3, Eif3e, Hnrnpd, Hnrnpf, Hnrnpu, Pabpc1, Polr2b, Srrm1, Siahbp1, Slbp, Snrpf, Sfrs11	3.16	16	4.3E-04	2.6E-01
**Protein transport**<br>Akap2, Arfgap1, Abca1, Rab1b, Sec23a, Sec61a1, Copg, Copz1, Kpna3, Mgea5, Nedd4, Nasp, Nupl1, Ramp2, Rtp4, Scamp5, Snx16, Snx27, Synj2bp, Snap29, Stx18, Stx6, Timm13, Vps4a, Vps45	2.24	24	3.4E-03	5.0E-01
**Nuclear mRNA splicing**<br>Bcl2l2, Ccar1, Cpsf3, Hnrnpf, Hnrnpu, Polr2b, Srrm1, Snrpf, Sfrs11	2.18	9	6.5E-03	5.3E-01
**Regulation of transcription**<br>Abca1, Crebbp, Ddit3, Fos, Fkbp1a, Klf13, Ndfip2, Smarca4, Yaf2, Anapc11, Bckdha, Arpp19, Cdk7, Csrnp2, Hif1a, Irak1, Mif, Med1, Mkl1, Mgea5, Nedd4, Neurod1, Nfia, Ncor1, Ncoa6, Nfyc, Nolc1, Otx2, Pbx1, Psmd1, Pias1, Stk11, Srf, Tbl1x	1.92	23	5.0E-03	5.8E-01
**Fatty acid transport**<br>Ace, Abcc1, Bdkrb2, Slc27a4, Slco1a5	1.47	5	2.3E-02	6.6E-01
**Regulation of carbohydrate metabolism**<br>Pfkfb2, Arpp19, Mif, Mgea5, Stk11	1.45	5	2.3E-02	6.6E-01
**Nucleotide binding**<br>Pfkfb2, Arl13b, Actr2, Abca1, Abcd2, Atp2a2, Ddx49, Elavl3, Ephb3, Grk6, Gtpbp6, Rab1b, Rev3l, Rbm15b, Raly, Rnd3, Smarca4, Smarca5, Ugp2, Upf3a, Acadvl, Csnk2a2, Cdc2l5, Chd2, Cdk10, Cdk7, Dld, Dclk2, Hnrnpd, Hnrnpf, Ide, Irak1, Kif1a, Lace1, Mars, Mast3, Mapk10, Nolc1, Pspc1, Fars2, Pde4d, Pabpc1, Polr2b, Rtkn, Rbs24, Stk11, Stk2r, Stk39, Siahbp1, Abcc1, Ddx27, Hsp90ab1, Spast, Sfrs11, Tars2, Trpm7, Ttl, Erbb4, Vrk1, Vps4a, Hars2l	1.44	60	1.6E-02	8.4E-01
**Potassium channel complex**<br>Cacna1a, Kctd1, Kcnk2, Kcnma1, Kcnd3, Kcnc2, Kcna6, Kcne2, Trpm7	1.41	7	2.0E-03	5.8E-02
**Macromolecular complex assembly**<br>Akap2, Abca1, Fkbp1a, Taf9b, Cacna1a, Cdk7, Eif3d, Hip1, Ide, Irak1, Mif, Med1, Nrco6, Polr2b, Kcnma1, Prlr, Pias1, Stk11, Hist2h2aa3, Snrpf, Spast	1.41	21	2.3E-02	6.6E-01
**Vesicle coating**<br>Arfgap1, CopG, Copz1, Hip1, Synj2bp	1.39	5	5.2E-03	5.6E-01
**DNA-dependent transcription**<br>Ddit3, Fos, Taf9b, Cdk7, Gtf3c6, Med1, Ncoa6, Polr2b	1.30	8	4.1E-02	7.4E-01

Supplementary Table 4. Selected literature. The role and impact of estrogen receptor beta signaling in the hippocampal formation with special emphasis on learning, memory, synaptic plasticity, depression, anxiety, fear, neuroprotection and neurogenesis.

**Learning/Memory/Synaptic plasticity**			
Activation of estrogen receptor beta regulates synaptic plasticity and improves memory	Liu, F et al.	Nature Neuroscience	11:334-343, 2008
Estrogen receptor alpha and beta specific agonists regulate expression of synaptic proteins in rat hippocampus	Waters, EM et al.	Brain Research	1290:1-11, 2009
Estradiol and ERbeta agonists enhance recognition memory, and DPN, an ERbeta agonist, alters brain monoamines	Jacome, L F	Neurobiol Learn Mem	94:488-498, 2010
Role of estrogen receptor alpha and beta expression and signaling on cognitive function during aging	Foster, TC	Hippocampus	22:656-669, 2012
Estradiol acts via estrogen receptors alpha and beta on pathw ays important for synaptic plasticity in the mouse hippocampal formation	Spencer-Segal, J L et al.	Neuroscience	202:131-146, 2012
Oestradiol-induced synapse formation in the female hippocampus: roles of oestrogen receptor subtypes	Zhou, L et al	J Neuroendocrinol	26:439-477, 2014
Bidirectional modulatory effect of 17beta-estradiol on NMDA receptors via ERalpha and ERbeta in the dentate gyrus of juvenile male rats	Tanaka, M and Sokabe, M	Neuropharmacology	75:262-273, 2013
Signaling mechanisms involved in the acute effects of estradiol on 5-HT clearance	Benmansour, S et al.	Int J Neuropsychopharmacol	17:765-777, 2014
Estradiol responsiveness of synaptopodin in hippocampal neurons is mediated by estrogen receptor beta	Fester, L et al.	J Steroid Biochem Mol Biol	138:455-461, 2013
Estradiol acutely potentiates hippocampal excitatory synaptic transmission through a presynaptic mechanism	Smejkalova,T & Woolley, C	J Neuroscience	30:16137-48, 2010
Estrogen receptor (ER) subtype agonists alter monoamine levels in the female rat brain	Lubbers, L S et al.	J Steroid Biochem Mol Biol	122:310-317, 2010
Estradiol targets synaptic proteins to induce glutamatergic synapse formation in cultured hippocampal neurons: critical role of estrogen receptor-alpha	Jelks, KB et al.	J Neuroscience	27:6903-6913, 2007
Androgens' effects to enhance learning may be mediated in part through actions at estrogen receptor-beta in the hippocampus	Edinger, KL and Frye, CA	Neurobiol Learn Mem	87:78-85, 2007
Increased estrogen receptor beta expression correlates w ith decreased spine formation in the rat hippocampus	Szymczak, S et al.	Hippocampus	16:453-463, 2006
Contribution of estrogen receptor subtypes, ERa, ERb, and GPER1 in rapid estradiol-mediated enhancement of hippocampal synaptic transmission in mice	Kumar, A et al	Hippocampus	25:1556-66.2015
Estrogen receptor-selective agonists modulate learning in female rats in a dose- and task-specific manner.	Pisani SL, et al.	Endocrinology	157:292-303, 2016
Re-Opening the Critical Window for Estrogen Therapy.	Bean, LA et al.	J Neuroscience	35:16077-93, 2015
**Depression/anxiety/synaptic plasticity**			
Administration of estrogen receptor beta-specific selective estrogen receptor modulators to the hippocampus decrease anxiety and depressive behavior of ovariectomized rats	Walf, AA and Frye,CA	Pharmacol Biochem Behavor	86:407-414, 2007
Antisense oligodeoxynucleotides for estrogen receptor-beta and alpha attenuate estradiol's modulation of affective and sexual behavior, respectively	Walf, AA et. Al.	Neuropsychopharmacology	33:431-440, 2007
Rapid and estrogen receptor beta mediated actions in the hippocampus mediate some functional effects of estrogen	Walf, AA and Frye,CA	Steroids	73:9-10, 2008
Selective estrogen receptor-beta (SERM-beta) compounds modulate raphe nuclei tryptophan hydroxylase-1 (TPH-1) mRNA expression and cause antidepressant-like effects in the forced sw im test	Clark, J A et al.	Neuropharmacology	63:151-1063, 2012
Effect of oestrogen receptor alpha and beta agonists on brain N-methyl-D-aspartate receptors	Morissette, M et al	J Neuroendocrinol	20:1006-1014, 2008
Estrogen, predominantly via estrogen receptor alpha, attenuates postpartum-induced anxiety- and depression-like behaviors in female rats	Furuta, M et al.	Endocrinology	154:38017-3816, 2013
Kalirin-7, an important component of excitatory synapses, is regulated by estradiol in hippocampal neurons	Ma, XM et al.	Hippocampus	21:661-677, 2011
Oestrogen receptor beta is involved in the actions of oestrogens in the brain for affective behaviour, but not trophic effects in peripheral tissues	Walf, AA	J Neuroendocrinol	22:141-151, 2010
**Contextual fear**			
Estrogen modulates sexually dimorphic contextual fear extinction in rats through estrogen receptor beta	Chang,YJ et al.	Hippocampus	11:1142-1150, 2009
**Neuroprotection/neurogenesis**			
Ovariectomy and subsequent treatment w ith estrogen receptor agonists tune the innate immune system of the hippocampus in middle-aged female rats	Sárvári, M et al.	Plos One	9:e88540, 2014
Age-dependent Effects of 17beta-estradiol on the dynamics of estrogen receptor beta (ERbeta) protein-protein interactions in the ventral hippocampus	Mott, NN et al.	Mol Cell Proteomics	13:760-779, 2014

Periodic Estrogen Receptor-Beta Activation: A Novel Approach to Prevent Ischemic Brain Damage	Cue, L et al	Neurochem Res	2014 (epub)
De novo synthesized estradiol protects against methylmercury-induced neurotoxicity in cultured rat hippocampal slices	Yamazaki, T et al.	PLoS One	8:e55559, 2013
Estrogen receptor beta treats Alzheimer's disease	Tian, Z et al.	Neural Regen Res	8:420-426,2013
Selective oestrogen receptor modulators differentially potentiate brain mitochondrial function	Irw in, RW et al.	J Neuroendocrinol	24:236-248, 2012
Neuroprotective role of estradiol against neuronal death induced by glucose deprivation in cultured rat hippocampal neurons	Hernandez-Fonseca, K et al.	Neuroendocrinology	96:41-50,2012
A priming role of local estrogen on exogenous estrogen-mediated synaptic plasticity and neuroprotection	Chamniansaw at, S and Chongthammakun, S	Exp Mol Med	44:403-411, 2012
Adult hippocampal cell proliferation is suppressed w ith estrogen w ithdraw al after a hormone-simulated pregnancy	Green, A.D and Galea, LA	Horm Behav	54:203-211, 2008
Neurosteroid estradiol rescues ischemia-induced deficit in the long-term potentiation of rat hippocampal CA1 neurons	Dai, X et al.	Neuropharmacology	52:1124-1138, 2007
Activation of estrogen receptor alpha increases and estrogen receptor beta decreases apolipoprotein E expression in hippocampus in vitro and in vivo	Wang, JM et al.	Proc Natl Acad Sci U S A	103:16983-16988, 2006
Both estrogen receptor alpha and estrogen receptor beta agonists enhance cell proliferation in the dentate gyrus of adult female rats	Mazzucco, CA et al.	Neuroscience	141:1793-1800, 2006
Neuroprotection by ovarian hormones in animal models of neurological disease	Hoffman, GE et al.	Endocrine	29:217-231, 2006
Estrogen can act via estrogen receptor alpha and beta to protect hippocampal neurons against global ischemia-induced cell death	Miller, NR et al.	Endocrinology	146:3070-3079, 2005
Estrogen receptor subtypes alpha and beta contribute to neuroprotection and increased Bcl-2 expression in primary hippocampal neurons	Zhao, L et al.	Brain Research	1010:22-34, 2004

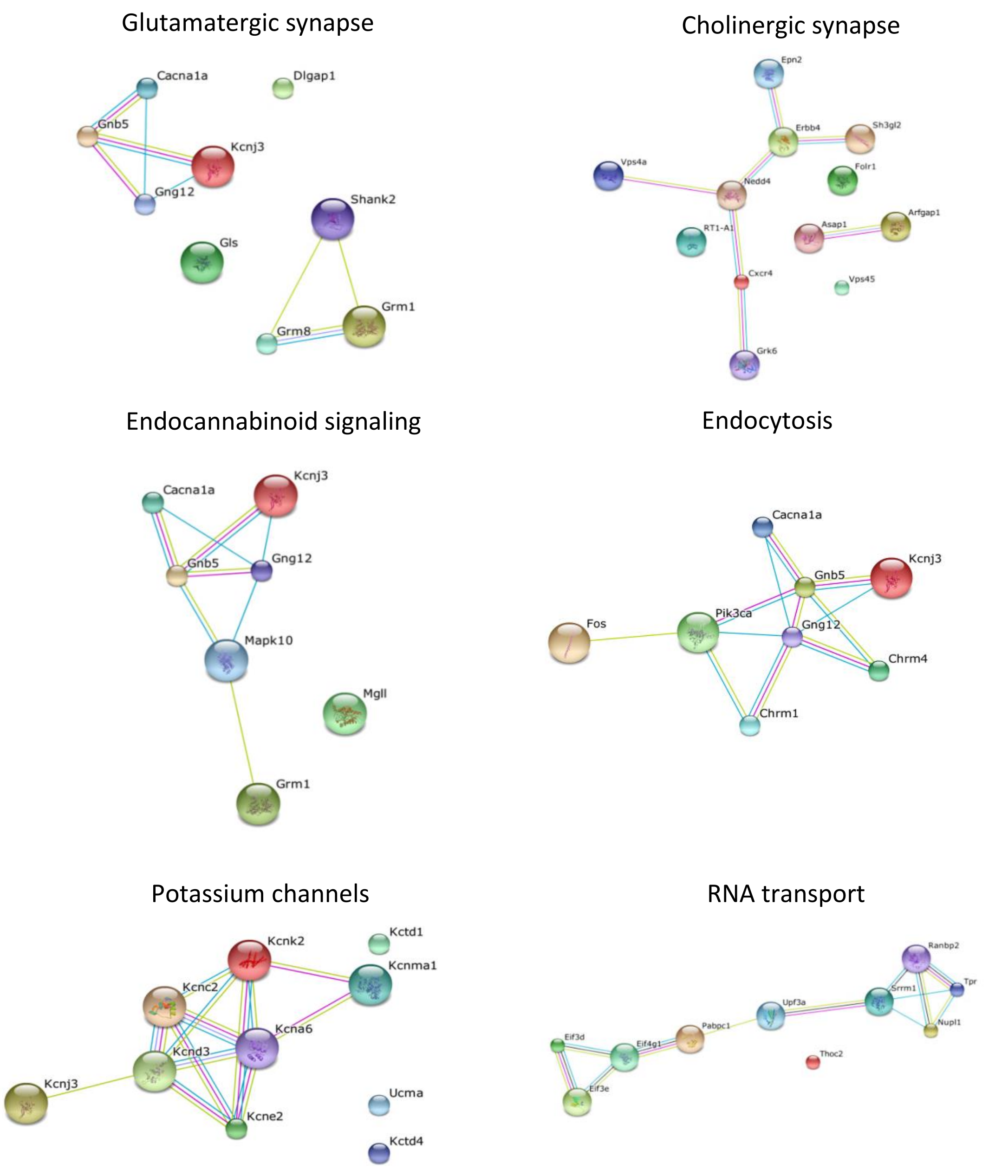

Supplementary Figure 1. Visualization of protein clusters encoded by DPN-regulated genes. The functional GO terms were generated by the KEGG pathway analysis (**Table 2**) and its members were plotted by the STRING program.

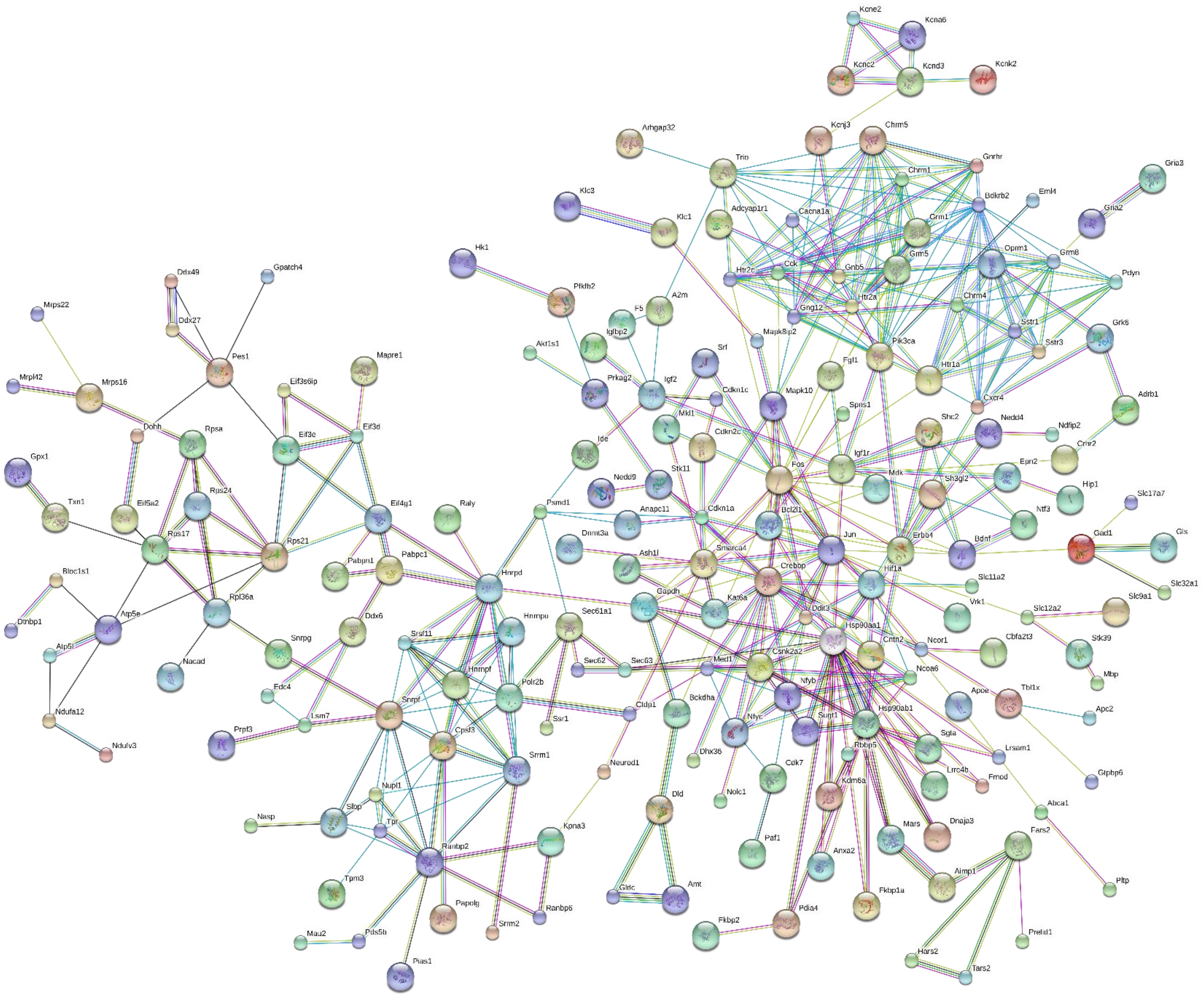

Supplementary Figure 2. Predicted interactions among proteins encoded by DPN-regulated genes. DPN regulation was determined by microarray and real-time PCR studies. STRING 10 program was applied to predict interactions. Disconnected nodes and clusters with less than 4 interacting protein members have been omitted from the chart. Confidence level value: 0.72.
